# Nestin‐expressing cell types in the temporal lobe and hippocampus: Morphology, differentiation, and proliferative capacity

**DOI:** 10.1002/glia.23211

**Published:** 2017-09-19

**Authors:** Joan Liu, Cheryl Reeves, Thomas Jacques, Andrew McEvoy, Anna Miserocchi, Pamela Thompson, Sanjay Sisodiya, Maria Thom

**Affiliations:** ^1^ Department of Clinical and Experimental Epilepsy UCL Institute of Neurology, Queen Square London WC1N 3BG United Kingdom; ^2^ Divisions of Neuropathology National Hospital for Neurology and Neurosurgery, Queen Square London WCN1BG UK; ^3^ Department of Biomedical Sciences University of Westminster London W1W 6UW United Kingdom; ^4^ Department of Neuropathology UCL Institute of Child Health and Great Ormond Street Hospital for Children London United Kingdom; ^5^ Neurosurgery at the National Hospital for Neurology and Neurosurgery, Queen Square London WCN1BG United Kingdom; ^6^ The Chalfont Centre for Epilepsy, Chesham Lane, Chalfont St Peter Buckinghamshire SL9 0RJ United Kingdom; ^7^ Department of Neuropsychology National Hospital for Neurology and Neurosurgery, Queen Square London WCN1BG UK; ^8^ Department of Neurology National Hospital for Neurology and Neurosurgery, Queen Square London WCN1BG UK

**Keywords:** hippocampal sclerosis, nestin, radial glia, temporal lobe epilepsy, white matter

## Abstract

Nestin is expressed in immature neuroepithelial and progenitor cell types and transiently upregulated in proliferative neuroglial cells responding to acute brain injury, including following seizures. In 36 temporal lobe (TLobe) specimens from patients with TLobe epilepsy (age range 8–60 years) we studied the number, distribution and morphology of nestin‐expressing cells (NEC) in the pes, hippocampus body, parahippocampal gyrus, amygdala, temporal cortex and pole compared with post mortem control tissues from 26 cases (age range 12 gestational weeks to 76 years). The proliferative fraction of NEC was evaluated in selected regions, including recognized niches, using MCM2. Their differentiation was explored with neuronal (DCX, mushashi, βIII tubulin, NeuN) and glial (GFAP, GFAPdelta, glutamine synthetase, aquaporin4, EAAT1) markers, both in sections or following culture. Findings were correlated with clinical parameters. A stereotypical pattern in the distribution and morphologies of NEC was observed, reminiscent of patterns in the developing brain, with increased densities in epilepsy than adult controls (*p* < .001). Findings included MCM2‐positive radial glial‐like cells in the periventricular white matter and rows of NEC in the hippocampal fimbria and sulcus. Nestin cells represented 29% of the hippocampal proliferative fraction in epilepsy cases; 20% co‐expressed βIII tubulin in culture compared with 28% with GFAP. Significant correlations were noted between age at surgery, memory deficits and nestin populations. TLobe NEC with ongoing proliferative capacity likely represent vestiges of developmental migratory streams and resident reactive cell populations of potential relevance to hippocampal epileptogenesis, TLobe pathology, and co‐morbidities, including memory decline.

**Abbreviations**
Aq4: aquaporin 4
CA: Cornu Ammonis
DCX: doublecortin
DG: dentate gyrus
EAAT1: exaitatory amino acid transporter 1
FZ: fimbria zone
GFAP: glial fibrillary acidic protein
GS: glutamine synthetase
HB: hippocampus body
HS: hippocampal sclerosis
Iba1: ionized calcium‐binding adapter molecule 1
MCM2: minichromosomal maintenance 2
NEC: nestin expressing cells
NeuN: Neuronal protein
PDGFRβ: platelet derived growth factor receptor beta
PHG: parahippocampal gyrus
PM: post mortem
PVWM: periventricular white matter
RMS: rostral migratory stream
SGZ: subgranular zone of dentate gyrus
SPL: subpial layer
SVZ: subventricular zone of the lateral ventricles
TLE: temporal lobe epilepsy
TLobe: temporal lobe
Tpole: temporal pole
WM: white matter
ZnT3: zinc transporter 3

## 1 INTRODUCTION

It is well established that the adult human brain retains regenerative neurogenic potential, which may be influenced by various physiological and pathological processes, including seizures (Jessberger & Parent, 2015). Recognized neurogenic niches include the subgranular zone (SGZ) of the hippocampus and the subventricular zone (SVZ) around the lateral ventricle (Eriksson et al., 1998; Goncalves, Schafer, & Gage, 2016; Levison & Goldman, 1997; Lim & Alvarez‐Buylla, 2016). Nestin is an intermediate filament protein widely used as a marker for proliferating neuroepithelial and progenitor cells (Lindqvist, Wistbacka, & Eriksson, 2016; von Bohlen und Halbach, 2011) when its expression is spatially and temporally regulated (Thomsen, Pallesen, Daugaard, Borglum & Nielsen, 2013); it is also transiently upregulated in proliferating neuroglial cells following brain injury (Buffo et al., 2008; Goc, Liu, Sisodiya, & Thom, 2014)

Hippocampal sclerosis (HS) is the commonest pathology encountered in patients with temporal lobe epilepsy (TLE) (Blumcke et al., 2013; Thom, 2014). As progenitor cell types reside in the adult hippocampus, altered rates of neurogenesis and gliogenesis, possibly influenced by seizures, are likely to be relevant to both histological alterations and clinical manifestations of this pathology. For example functional alteration in the distribution and maturity of astroglia (Pekny et al., 2016) contribute to hippocampal epileptogenesis (Bedner et al., 2015) as well co‐morbidities including memory dysfunction (Hattiangady & Shetty, 2010; Sajja,, Hlavac, & VandeVord, 2016) which are prevalent in TLE (Helmstaedter & Elger, 2009).

The aim of this study was to explore the distribution and morphology of nestin‐expressing cell (NEC) populations in HS compared with control groups, in recognized niches as well as other temporal lobe (TLobe) regions. In addition, we aimed to explore their proliferative capacity, evidence to support neuronal versus glial maturation and their relation to the underlying histopathology and clinical features, in particular, memory dysfunction.

## 2 MATERIALS AND METHODS

### 2.1 Case selection and regions studied

The study has ethical approval. Brain samples from 62 cases were obtained through the Epilepsy Society Brain and Tissue Bank at UCL Institute of Neurology, Great Ormond Street Hospital NHS Trust, MRC‐Wellcome Trust Human Developmental Biology Resource and MRC Sudden Death Brain Bank, Edinburgh. These included surgical specimens from 36 adult or pediatric patients undergoing treatment for TLE with a diagnosis of (a) HS, (b) No‐HS, or (c) lesional hippocampal pathologies. Six adult post mortem (PM) epilepsy cases with HS were also included in the study. PM brain samples from five developmental fetal controls, and 15 normal adult controls were used for comparison (Table 1; see Supporting Information Table 1 for full details of all cases). HS was classified according to International League Against Epilepsy (ILAE) guidelines (Blumcke et al., 2013). Standard anterior temporal lobectomy was performed on all surgical cases including six anatomical regions: (a) temporal neocortex (TLobe) [superior temporal gyrus to fusiform gyrus, 1.5 cm caudal to temporal pole (TPole)], (b) TPole, (c) mid‐hippocampal body (HB), (d) pes hippocampus (PES), (e) parahippocampal gyrus (PHG), and (6) amygdala. In PM cases, comparable regions were examined and both hemispheres in four PM cases with bilateral HS.

**Table 1:** Clinical and pathology details of cases and control groups (Further detail of each case is available in Supporting Information Table S1)

Group	Tissue type	Number of cases	Predominant pattern of HS in body	Age (at surgery or death); mean (range); Age of onset of epilepsy Range) Years	Gender	Laterality of HS	Regions examined
Adult epilepsy TLE/HS EA1‐22	Surgical fixed	22	Type 1 HS (14) No HS (7) Type 2 (1∼)	38.5 (22–60); 13.87 (1.3–35)	10F: 12M	12L: 10R	HB,PES,PHG,TPole, TLobe, Amyg
Adult epilepsy TLE/HS EAC 1‐6	Surgical/cell culture	6	Type 1 HS	42 (29–52); 15 (2–15)	3F: 3M	4L: 2R	HP (inclusive of HB and PES), TLobe (and Amyg in two cases)
Adult epilepsy TLE/LEAT EAT1–3	Surgical fixed	3	Type 1 HS (1) Type 2 HS (1) Type 3 HS (1)	32.6 (27–46); 13 (7–20)	2F: 1M	3L	HB,PES,PHG,TPole, TLobe, Amyg
Pediatric epilepsy TLE/HS EP1‐5	Surgical fixed	5	ILAE type 1 HS only	12.2 (8–15); 3.9 (0.6–7.5)	2F: 3M		HB, TLobe
Fetal controls	Developmental controls	5	No pathology	12–13pcw	‐	N/A	Whole brain including TLobe and hippocampus
Adult epilepsy EPM1‐6	PM	6	ILAE Type 1 HS	57.8 (26–76)	3F: 3M	Bilateral in 4 cases	HB, PHG (Both hemispheres included in four cases)
Adult non‐epilepsy controls C1‐15	PM	15	No HS	49.5 (28–75)	6F: 9M	N/A	HB, PHG, Amyg, Cortex (Both hemispheres in five cases)

EA, epilepsy adult; EP, epilepsy pediatric; EPM, adult epilepsy PM; C, PM control; N/A, not applicable; PCW, post‐conception week; HB, hippocampal body; PES, pes hippocampus; PHG, parahippocampal gyrus; TPole, temporal pole; TLobe, temporal lobe; Amyg, amygdala. ∼The single case of Type 2 HS was not used in any quantitative analysis; HS, hippocampal sclerosis; ILAE, International League against epilepsy; TLE, temporal lobe epilepsy; LEAT, Low‐grade; epilepsy‐associated tumor.

### 2.2 Immunohistochemistry

Immunochemistry for nestin was carried out on 5 µm‐thick formalin‐fixed, paraffin‐embedded sections from all regions in each of the cases. Comparative immunohistochemistry or double immunofluorescent labeling was carried out for: immature and mature neuronal markers (doublecortin (DCX), mushashi, NeuN), astroglial lineage markers (GFAP, GFAP‐delta isoform (GFAPδ), glutamine synthetase (GS), aquaporin 4 (Aq4), excitatory amino acid transporter 1 (EAAT1), microglia (Iba1), oligodendroglial lineage (olig2), endothelium (CD34), pericytes (PDGFRβ), mTOR activation (pS6), cell proliferation (MCM2), and mossy fiber sprouting (ZnT3), using standard protocols (Table 2, and Supporting Information Methods).

**Table 2:** Antibodies and protocols for immunohistochemical studies

Antibodies, Clone, Code	Immunogen or target epitope	Labeled cell or protein type	Antibody supplier; dilution, incubation period (min)	Pre‐treatment (min)
**NEURONAL LINEAGE**				
**Anti‐Nestin**10C2, AB22035	150 aa recombinant fragment from human nestin	Immature progenitors, glia, endothelial cells	Abcam; 1:1000, ov	H‐3301 (12)
**Anti‐Dcx**4604	Synthetic peptide to human Dcx	Immature progenitors, neuroblasts, neuroglia	Cell Signaling; 1:250, ov	SC (12)
**Anti‐Musashi**AB5977	Synthetic peptide of musashi (aa 5‐21)	Immature progenitors, neuroglia	Millipore; 1:500, ov	H‐3301 (12)
**Anti‐NeuN**A60, MAB377	Purified cell nuclei from mouse brain	Nuclei of most neurons, some proximal dendrites	Millipore; 1:2000, 15	ER1 (20)
**Anti‐Synaptophysin**IR660	Recombinant fragment to human synaptophysin (C‐term.)	Neurons with synaptic vesicles	DAKO; 1:100, 20	ER2 (20)
**Anti‐MAP2**HM‐2, M4403	Rat MAPs	Neurons	Sigma; 1:1500, ov	H‐3301 (12)
**Anti‐ZnT3**197–003	Recombinant protein of mouse ZnT3 (aa. 2‐75)	Synaptic vesicle located zinc‐transporter	Synaptic Systems; 1:10,000, ov	H‐3301 (12)
**ASTROGLIA AND FUNCTIONAL MARKERS**				
**Anti‐GFAP**Z0334	GFAP isolated from cow spinal cord	Astrocytes, some ependymal cells	DAKO; 1:2500, 20	ENZ1 (10)
**Anti‐GS**GS‐6, MAB302	GS purified from sheep brain	Astrocytes	Millipore; 1:500, ov	H‐3301 (12)
**Anti‐AQ4**A5971	Recombinant fusion protein to residues 249–323 of rat AQ4	Water channels expressed mainly by astrocytes	Sigma Aldrich; 1:500 ov	H‐3301 (12)
**Anti‐EAAT1/GLAST** AB181036	Synthetic peptide within human EAAT1 (aa. 150‐25)	Glutamate and aspartate transporters expressed mainly by astrocytes	Abcam; 1:800 ov	H‐3301 (12)
**MTOR PATHWAY**				
**Anti‐pS6**D68F8, 5364	Residues surrounding Ser240&244 of human S6	Cells active in mTORC1 pathway	Cell Signaling; 1:1000, ov	H‐3301 (12)
**Anti‐pS6**91B2, 4857	Residues surrounding Ser235&236 of human S6	Cells active in Ras‐MAPK mTOR‐independent pathway	Cell Signaling; 1:250. ov	H‐3301 (12)
**OLIGODENDROGLIAL AND MYELIN**				
**Anti‐Olig2**AB9610	Recombinant mouse Olig‐2	Oligodendroglia	Millipore; 1:300, ov	SC (12)
**SMI 94**SMI‐94R	Human myelin basic protein (70–89 aa)	Myelinated fibers in WM	DAKO; 1:2000, 20	ENZ1 (10)
**Microglia, endothelium/pericytes and cell cycle**				
**Anti‐Iba1**019‐19741	Synthetic peptide to C‐terminus of Iba1	Macrophage and microglia	WAKO; 1:1000, ov	H‐3300 (12)
**Anti‐PDGFRβ**Y92, AB32570	Synthetic peptide within human PDGFRβ (aa 1050 to the C‐terminus)	NG2/oligodendroglial precursor cell types, pericytes	Abcam; 1:1000, ov	H‐3300 (12)
**Anti‐CD34**QBEnd10, IR632	Endothelial cell membranes obtained as vesicles from human placenta	Endothelial cells	DAKO; 1:25, ov	H‐3300 (12)
**Anti‐Mcm2**46/BM28, 610700	Human BM23 aa.725–888	Proliferating cells	BD; 1:900, ov	H‐3301 (12)

All primary antibodies were incubated at room temperature unless otherwise stated. aa, amino acids; AQ4, aquaporin 4; Dcx, doublecortin; EAAT1, excitatory amino acid transporter 1; GFAP, glial fibrillary acidic protein; GLAST, glutamate aspartate transporter; GS, glutamate synthetase; iba1, ionized calcium‐binding adapter molecule 1; MAP, microtubule associated protein; mTOR, mammalian target of rapamycin; NeuN, neuronal nuclei; Olig2, oligodendrocyte transcription factor 2; ov, overnight at 4°C; PDGFRβ, platelet derived growth factor receptor beta; pS6, phospho‐S6 ribosomal protein. Antigen retrieval buffers: ENZ1, Leica Bond enzyme concentrate and diluent; ER1, Leica Bond citrate‐based buffer; ER2, Leica Bond EDTA‐based buffer; H‐3301, Vector's Tris‐based buffer pH 9.0; H‐3300, Vector's citrate‐based buffer pH 6.0; SC, Sodium citrate buffer, pH 6.0. Suppliers: Abcam plc., Cambridge, UK; BD Transduction Lab., Oxford, UK; Cell Signaling Tech., Boston, US; DAKO, Cambridgeshire, UK; Leica, Milton Keynes, UK; Merck‐Millipore‐Sigma Aldrich, Hertfordshire, UK; Santa Cruz Biotechnology Inc., Heidelberg, Germany; Sternberger, Maryland, US; Swant, Marly, Switzerland; Synaptic systems GmBH, Goettingen, Germany; WAKO Pure Chemical Industries Ltd., Osaka, Japan.

### 2.3 Quantitative Analysis and Regions of Interest

**Figure 1:**
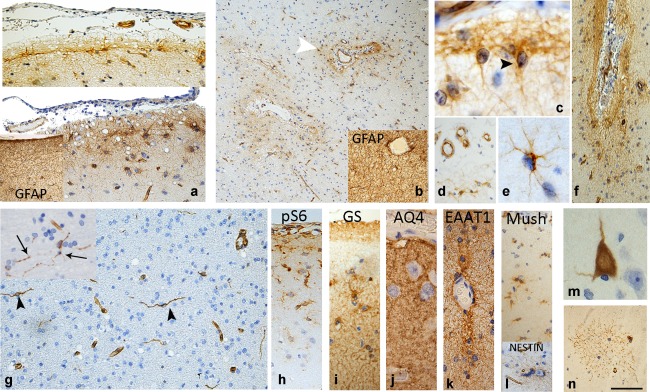
NEC types in temporal neocortex and TPole. (a) SPL of the temporal neocortex. Top, one case showing a continuous line of NEC. Bottom, more intermittent multipolar NEC are noted; the inset shows GFAP stain in the same case with more extensive cells and process in the SPL and layer I region. (b) WM: NEC show a restricted location to the perivascular regions (white arrowhead) in comparison to GFAP labeling in the same case (inset) which shows a diffuse astrogliosis. (c) NEC morphology: NEC in the SPL often display a triangular shaped soma (arrowhead) sitting in the “Chaslins” subpial band with both horizontal and radial processes. In PM cases (d) less frequent NEC in the SPL were observed, despite strong labeling of endothelium. (e).Mulitpolar NEC: these were the predominant morphological cell type in both the cortex and WM. (f) Sulcal NEC and fibers were typically more densely packed than in the gyral crests. (g) Inferior temporal gyrus WM: elongated nestin‐positive threads and bipolar cells were present (arrowheads); the inset shows fine branching and *varicous* or beaded processes of these cells (arrows). (h) MTOR activation/pS6 Prominence of labeling of cells in the subpial region and cortical layer I. (i) GS: A marker of functionally mature astrocytes shows labeling in the Chaslins subpial band, patchy labeling in layer I and diffuse and uniform pancortical labeling of astrocytes and processes in the cortex. (j) Aq4 shows specific and dense labeling of glial processes in the cortex and foot processes around vessels. (k) EAAT1 shows labeling of astroglial cells including around vessels. (l) Mushashi: Cytoplasmic labeling of clusters and doublets of small multipolar cells mainly in layer I is observed; inset shows similar cluster of NEC. (m) Neuronal labeling: Rare cortical pyramidal cell labeled with nestin in the TPole. (n) Occasional finding was a ‘tuft‐like’ pattern of nestin processes in the temporal neocortex. Bar = 120 microns in (a,f,d,g,h–l); 50 microns in (e,m); 300 microns in (b), approximated based on original magnifications [Color figure can be viewed at wileyonlinelibrary.com]

**Figure 2:**
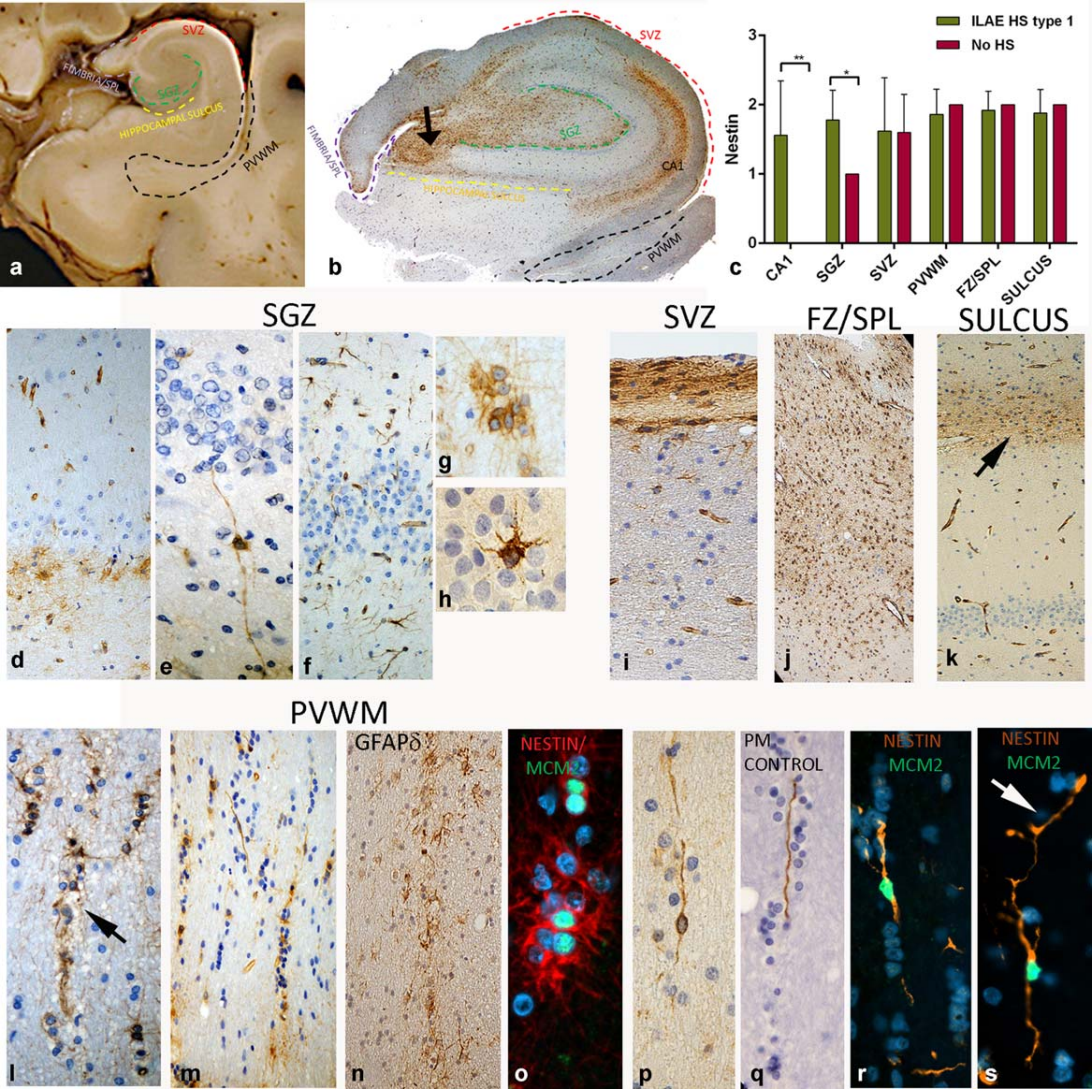
NEC in the HB. (a) Zones on hippocampus: Regions of the hippocampus used in qualitative and quantitative evaluation. (CA1, CA4, and fimbria (F) indicated on image of PM case). Region 1 (dashed red line) SVZ, the region underlying the lateral ventricle wall; Region 2 (dashed black line) PVWM, the region surrounding the tail of the temporal horn of the lateral ventricle, extending toward the PHG; Region 3 (dashed purple line) FZ/SPL; Region 4 (dashed yellow line) Hippocampus sulcus (or fissure), the WM adjacent to the sulcus (arrow) between the dentate gyrus and subiculum. (b) Nestin labeling in hippocampal regions: The regions of nestin labeling (SVZ, Fimbria/SPL, PVWM, SGZ and hippcocampal sulcus) are indicated on this low power view. At this magnification prominent labeling with nestin is most visible in SGZ and CA4 extending towards to SPL (arrow). In addition in this case of Type 1 HS dense labeling is noted in the CA1 region. (c) Bar chart of semi‐quantitative evaluation of mean NEC densities in hippocampal subregions between cases with HS (ILAE Type 1) and with No‐HS. Significant differences were noted for CA1 and CA4 regions only (**p < .001, *p < .01). SGZ: (d) A prominent pattern was a row of multipolar NEC in the immediate SGZ; (e) Occasional bipolar NEC traversed the dentate gyrus; (f) Multipolar NEC were generally less frequent in the molecular layer of the dentate gyrus; (g) Clusters of NEC in SGZ and CA4 were particularly prominent in pediatric HS cases; (h) Occasional granule cell neurons were nestin positive. (i) SVZ: A prominent band of NEC with cells extending into the underlying WM. (j) FZ/SPL: A typical finding was rows or streams of NEC extending down into the underlying WM. (k) Hippocampal sulcus: Rows of NEC forming a band were present in this region (arrow). PVWM: (l,m) Lines or chains of NEC formed rows often running in parallel with the tail of the temporal horn of the ventricle in a direction toward the PHG. (n) GFAP‐delta: Similar rows of cells were prominent with GFAPdelta immunostaining around the ventricular cavity in the PVWM. (o) Nestin/MCM2: double labeling with cell cycle marker MCM2 confirmed a proportion of the NEC forming rows of cells in the PVWM showed nuclear positivity. (p) Bipolar NEC: These were observed predominantly in the PVWM than other regions and often running in parallel with lines of small “oligodendroglial‐like” round nuclei. (q) Nestin threads: Elongated nestin‐positive, radial‐glial like fibers were also observed in the PVWM, often forming a central core with surrounding chains of small cells. (r) Nestin/MCM2: Bipolar nestin‐positive cells with nuclear MCM2^+^; (s) branch points and varicose process were observed (arrow). Bar = 500 microns in (a), 200 microns in B, 30 microns in (e,g–i,l,m,o–s), and 50 microns in (d,f,k,j,k); approximated based on original magnifications

The distribution and morphology of NEC was evaluated in regions of interest (ROIs) including known neurogenic niches: **TLobe** [subpial layer (SPL), cortex, and white matter (WM) in the middle temporal gyrus], **TPole** (SPL, layer I, WM), **HB** [Cornu Ammonis regions 1 and 4 (CA1, CA4)], SGZ of dentate gyrus, SVZ, fimbria and SPL of hippocampus (FZ/SPL), hippocampal sulcus, periventricular white matter (PVWM) of hippocampus, **PES** (SGZ, CA4, SPL, PVWM), **PHG** (cortex, WM), amygdala (all nuclei, WM and peri‐amygdala cortex) (Figures 1a,b and 2a). In each of these regions, a four‐point scale was used for the semi‐quantitative evaluation: (0) no NEC, (+) rare NEC, (++) few NEC with patchy distribution along layer/zone, (+++) frequent NEC with continuous/extensive distribution along the layer/zone.

In 16 surgical cases (15 adult and 1 pediatric surgical cases with and without HS) and 5 PM controls (see Supplementary Table 1), MCM2/nestin double‐labeled sections from HB, PES and Tlobe were quantified. In three Type 1 HS cases (EA3, EA4 and EA7) single and double‐labeled cells for MCM2 with olig2, GFAP or Iba1 co‐expression in HB ROI were quantified to characterize the relative proportions of MCM2 immuno‐positive proliferating cells (see Supporting Information Methods).

### 2.4 Cell Culture

To investigate the lineage of NEC *in vitro*, cells were cultured from 0.5 to 1.2 g of fresh tissue taken from the hippocampus sample (pes and HB including all subfields, WM and dentate gyrus), and the gray and WM of the TPole from six cases with mesial TLE and Type 1 HS (cases EAC1‐6). Up to 0.2 g of tissue from the amygdala was available for culture from two cases (EAC4‐5). Dissociated cells were cultured in MACS Neuro Medium (Miltenyi Biotec Ltd., Surrey, UK) supplemented with 1% MACS NeuroBrew‐21, 1% penicillin and streptomycin, 10 ng/ml EGF, 10 ng/ml bFGF, and 10% fetal bovine serum (detailed in Supporting Information Methods). After 4 weeks in culture, cells were immunolabeled for nestin and GFAP or βIII‐tubulin and immunolabeled cells were visualized using confocal scanning laser microscopy (LSM710; Zeiss, Germany). An average area of 45 ± 4 mm^2^ was imaged from each culture. Images were imported into the image analysis software Definiens Tissue Studio 3.6 and Definiens Developer x64 (Definiens AG; Munich, Germany) for automated quantification as previously described in (Liu et al., 2014b). Final results were expressed as percentages of NEC that co‐expressed GFAP or βIII‐tubulin, percentages of hoechst‐positive cells that expressed nestin and GFAP, βIII‐tubulin or both per culture. The average area of NEC from each region of each case was also recorded and the number of primary branches from each NEC, were manually counted.

### 2.5 Clinical Data

Age at onset and duration of epilepsy, seizure types and laterality of HS were obtained from the clinical records (Table 1 and Supporting Information Table S1). Results from standardized pre‐operative psychometric memory and naming tests and measures of memory decline at one year post‐operatively were also reviewed (Supporting Information Table S1 and Supporting Information Methods).

Statistical analysis was carried out between pathology groups, regions and zones and with clinical and psychometric data using non‐parametric tests with SPSS (IBM, California, USA; version 22); uncorrected *p* values of < .05 were regarded as significant. For cell culture data, non‐parametric (Kruskal‐Wallis and Spearman correlation) were used to determine whether the areas and the percentages of immunolabeled or co‐localized cells differed significantly between regions or correlated with age at surgery.

## 3 RESULTS

### 3.1 Nestin expression: Developmental control

In fetal brains of 12–14 gestational weeks, NEC and immunolabeled radial processes from these cells, were numerous in the SVZ of the lateral ventricle (Supporting Information Figure S1a,b) extending along the temporal horn, overlying the surface of the developing hippocampus (Supporting Information Figure S1c,d). Proliferating NEC formed rows and cords extending from the ventricular surface to the underlying, developing pyramidal cell layer of CA1 (Supporting Information Figure S1c,d) alongside radial nestin^+^ fibers (Supporting Information Figure S1e), bipolar NEC and small capillary channels (Supporting Information Figure S1a, inset). Of note, the subpial surface of the developing hippocampus, including the hippocampal sulcus anlage, showed a dense band of NEC, compared with less frequent NEC in the SPL of the developing neocortex (Supporting Information Figure S1a).

### 3.2 Nestin expression: Operated epilepsy cases

**T lobe**: Similar patterns of NEC regional distribution were noted across surgical cases. NEC were prominent in the SPL forming a continuous band of cells and processes (Figure 1a), particularly prominent in sulci (Figure 1f). NEC in the SPL often displayed triangular shaped somata with processes extending both horizontally and radially for short distances (Figure 1c), contributing to ‘Chaslin's’ band of gliosis. Single or small clusters of multipolar NEC were present in layer I (Figure 1a,d,e,l) with short branching processes (Figure 1e). In the WM, multipolar NEC occurred mainly in proximity to vessels (Figure 1b). In the inferior TLobe WM, bipolar NEC and thread‐like processes were prominent (Figure 1g). **T Pole**: Showed a similar morphology and distribution of NEC, with only occasional labeling of cortical neurons (Figure 1m). On semi‐quantitative analysis, NEC were significantly less frequent in the cortex (*p* < .0001) and WM (*p* < .005) than the SPL; there was no significant difference however between TLobe and TPole or between cases with Type 1 HS and No‐HS.

#### 3.2.1 Hippocampal body

Small multipolar NEC showed a stereotypical distribution pattern (Figure 2b), prominent in the SGZ, extending through the CA4 region, typically forming a continuous stream with the overlying fimbrio‐dentate sulcus (Figure 2b, arrow). NEC often formed a band along the basal layer of the granule cell layer (Figure 2d) and occasional bipolar cells with long processes extending through to the molecular layer (Figure 2e) and multipolar NEC in the molecular layer (Figure 2f) were noted. Clusters of NEC in SGZ were more frequent in pediatric HS cases (Figure 2g). Only rare labeling of granule cells was noted (Figure 1h). NEC were prominent in CA1 (Figure 2b) in type I HS cases (Figure 1b) but no labeling of hippocampal neurons seen. NEC populations were prominent in the SVZ, FZ/SPL of hippocampus, hippocampal sulcus and PVWM, with similar patterns in cases, regardless of the presence of HS or not (Figure 2i,j,k). NEC in these regions were often observed in rows or cords (Figure 2l,m), reminiscent of fetal brain (Supporting Information Figure S1). In the PVWM, particularly in the vicinity of the ventricle tail, bipolar NEC and elongated nestin^+^ fibers were visible alongside rows of cells (Figure 2p,q). Nestin^+^ fibers were occasionally finely branching and varicose (Figure 2r,s) and also observed in proximity to capillaries. Semi‐quantitative analysis of NEC showed significantly greater numbers in CA1 and SGZ regions in HS Type 1 than No‐HS cases (*p* < .01) (Figure 2c), but not for other hippocampal regions.

#### 3.2.2 PES hippocampus

**Figure 3:**
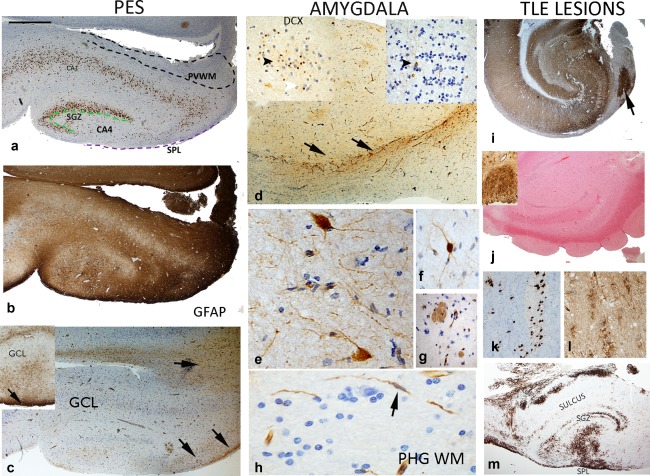
Nestin expression in the PES hippocampus, PHG, Amygdala and microscopic TLobe lesional pathologies. (a) Pes hippocampus: CA4, SGZ SPL and PVWM zones used for quantitative analysis indicated in this NeuN section; neuronal loss is apparent in CA4 corresponding to Type 3 HS. (b) GFAP shows extensive labeling of glial fibers through CA4 and the WM. (c) Nestin shows comparative restricted expression to the SPL (two arrows) and chains of cells and threads or fibers in the PVWM (single arrow); Inset shows another case with streams of cells from SPL into the CA4 region. (d) Amygdala: Chains of NEC are seen at the border of the amygdala in the vicinity of the paralaminar nucleus (arrows); Insets show DCX (left) and nestin expression (right) in the paralmainar nucleus with DCX showing labeling of small immature rest cells and scattered NEC with long processes (arrowhead). (e,f) Large NEC with morphology of neurons were more frequently noted in the amygdala than other temporal regions. (g) Frequent nestin^+^ thick fiber bundles traversed the amygdala. (h) PHG WM: Elongated nestin^+^ processes in the pes WM and biopolar cells (arrow). (i) Alveus heterotopia: Mature neuronal cells were present between the SPL and CA3 in continuity with the SPL in a patient without HS (shown in MAP2; further images in Supporting Information Figure S2a–d). (j) Subventricular nodules: Nestin+ cells (shown in inset) protrude into SVZ surface with evidence for some neuronal maturation, in a case with Type 2 HS (Further images in Supporting Information Figure S2e–h). (k) WM heterotopia: Islands of mature NeuN^+^ neurons associated with (l) NEC were present in the PVWM of the hippocampus and PHG (Further images in Supporting Information Figure S2m–o) (m) Low‐grade epilepsy‐associated tumors (LEAT): Prominent nestin and CD34^+^ (shown here) cells in the hippocampus SPL, sulcus and PVWM, remote from the main tumor lesion. (For i–m: see also Supporting Information Figure S2). Bar in (A–C,I,J,M), 500 microns, (d,k,l,g), 200 microns, and (e,h), 50 microns (approximate based on original image magnifications) [Color figure can be viewed at wileyonlinelibrary.com]

PES samples were available in 17 patients; In 5/11 Type 1 HS cases (based on HB), neuronal loss in the PES was restricted to CA4 region ((Figure 3a) and also in 4/6 of cases with No‐HS in the body. Granule cell dispersion was prominent and mossy fiber sprouting with ZnT3 staining indicating Type 3 pattern of HS (Blumcke et al., 2013) in the PES. NEC in the PES were primarily located in four regions (Figure 3c); the SGZ, CA4, the SPL, and the PVWM. Streams of NEC extended from the SPL surface of the PES into CA4 (Figure 3c, inset). Chains and rows of NEC, nestin^+^ fibers and bipolar cells were prominent in PES PVWM (Figure 3c). Semi‐quantitative analysis showed no significant difference in NEC populations in any region of the PES in relation to the HS type (as classified in either the PES or HB).

#### 3.2.3 PHG

PHG, comprising WM and cortex, was available in 14 cases. NEC were present in the cortex SPL. Semi‐quantitative analysis did not show significant differences from the TLobe cortex. Bipolar NEC, elongated nestin^+^ processes and rows of NEC were conspicuous in the WM, running perpendicular to the cortex in 12/14 cases (Figure 3h).

#### 3.2.4 Amygdala

Nestin labeling of scattered larger cells with neuronal morphology was frequently noted (Figure 3e,f), in addition to multipolar NEC and traversing, thick nestin^+^ fiber bundles (Figure 3g). Although in fragmented specimens it was not possible to identify amygdala nuclei with confidence, the paralaminar nucleus was identifiable by aggregates of small immature cells, a proportion showing nestin labeling (Figure 3d). In some cases, rows and streams of multipolar NEC, nestin^+^ fibers and capillary channels were also prominent at the border of this nucleus (Figure 3d). There was no correlation between semi‐quantitative analysis of NEC in the amygdala and patterns of HS.

#### 3.2.5 Other focal TLobe pathologies

Microscopic heterotopia. In case EA14 (Figure 3i; Supporting Information Figure S2a–d), islands of mature ‘heterotopic’ NeuN^+^ and MAP2^+^ neurons were present in the SVZ of the hippocampus in continuity with NEC lining the wall of the lateral ventricle; ZnT3^+^ immunopositive axons extended around these heterotopic neurons, indicating connection with mossy fibers. In case EA15 from a patient with a prior history of CNS infection, nodules of NEC were present along the SVZ with synaptophysin labeling (Figure 3j; Supporting Information Figure S2e–h). In case EA16 (Figure 3k,l; Supporting Information Figure S2i–l) islands and rows of small NEC in the PHG and PVWM were intermingled with mature neurones (MAP2^+^ and NeuN^+^) and as multipolar CD34^+^ cells. Low grade epilepsy associated tumor (LEAT). In three cases of CD34^+^ LEATs in the TLobe, small multipolar cells (nestin^+^/CD34^+^) showed stereotypical distributions in the HB involving the SPL, sulcus, SGZ and PVWM (Figure 3m; Supporting Information Figure S2p–s).

### 3.3 PM cases (epilepsy and controls)

In the six epilepsy PM cases with HS, there was an impression of fewer NEC overall compared with surgical HS, despite strong nestin capillary endothelial labeling (Figure 1d). In bilateral HS cases, similar patterns were seen in both hippocampi. NEC in the SVZ of the HB were present in 4/6 cases, including cases in their 70s (EPM1 and 4); in one case labeling of NEC in the FZ/SPL of the hippocampus was striking (case EPM4). NECs were less frequently present in CA1 or the SGZ of the hippocampus than surgical cases. Thread‐like nestin^+^ fibers were present in 2/6 cases in the PHG WM, but chains of NEC not apparent. Labeling of NEC in the cortex of the PHG was present in 1/6 cases.

In the PM controls, observations were similar to epilepsy PM cases, with NEC in SVZ of the HB in 7/15 cases, with one case (C5) also showing striking labeling in the FZ/SPL. NEC were noted in the SGZ in three cases, but not in CA1. Nestin^+^ threads and rare bipolar NEC were noted in the PHG in 3 cases, and NEC in SPL of the TLobe in 7/15 cases. Sections of amygdala in 6/15 showed NEC in the peri‐amygdalar WM, associated with nestin^+^ elongated threads in one case (C10). There was weak nestin labeling of fiber bundles running through the amygdala in one case (C11) but no neuronal labeling. Semi‐quantitative analysis showed that although SPL cortical NEC were more frequent in epilepsy PMs there were no significant differences with non‐epilepsy control PMs. There was no correlation across PM cases between the age at death and NEC densities.

### 3.4 Differentiation and maturation of NEC in fixed tissue sections

**Figure 4:**
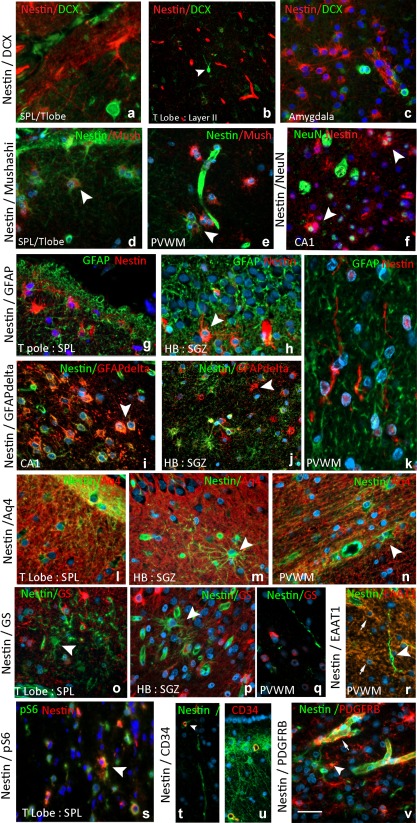
Differentiation and lineage of NEC from different regions. DCX: (a) The SPL of the TLobe shows a band of NEC and processes but DCX is confined to occasional small immature cells. (b) In cortical layer II occasional small tufted immature DCX+ neurons were seen (arrowhead) but nestin is confined to the endothelium. (c) In the amygdala paralaminar nucleus, small DCX and nestin positive cells form distinct, intermingled populations. Mushashi: (d) Small cells in layer I of the cortex co‐localized with nestin and (e) in the PVWM (arrowheads). (f). NeuN: Expression was mainly restricted to mature, large neurons but occasional expression in small NEC was observed (arrowheads). GFAP: (g) Expression was more extensive in all compartments than nestin and in this field of the SPL, NEC are mainly GFAP negative. (h) In the SGZ multipolar NEC co‐expressing GFAP were seen (arrowhead). GFAP‐delta: (i) There was more extensive co‐expression with nestin compared with GFAP, particularly in CA1 astrocytes (arrowhead) but (j) NEC‐negative cells were also observed. (k) Nestin‐positive bipolar cells and threads in the PVWM were GFAP (shown) and delta negative. Aq4: (l–n). Aq4 showed extensive labeling of astrocytic processes through all regions (shown here for the SPL, SGZ, and PVWM) but in all regions NEC lacking Aq4 expression were noted (arrowheads). GS: (o) In the SPL (shown here) and cortex numerous astroglial cell bodies were positive for GS forming an extensive plexus. A striking proportion of NEC were GS negative and also observed in the (p) SGZ (arrowhead) as well as (q) fibers. (r) Excitatory amino acid 1 (EAAT1) showed labeling of astroglial like cells (arrows), including perivascular foot processes; NEC in all regions including bipolar cells (arrowhead) were EAAT1 negative. pS6: (s) Small NEC show frequent co‐labeling with pS6, shown here in the SPL. CD34: (t) CD34 labeling was confined to the capillary endothelium in the PVWM and other regions including (u) the SVZ and not in NEC or elongated threads and bipolar cells. PDGFRbeta: (v) Labeled pericytes around nestin‐positive endothelium (arrow) were noted in addition to scatted small multipolar cells, a small number of which co‐labeled with nestin (arrowhead). Bar is equivalent to 35 microns (based on original magnifications)

Neuronal and glial markers were used to further characterize regional NEC in selected cases (Supporting Information Table S1). **Neuronal markers**: DCX labeled distinct cell populations with little overlap with NEC in the TLobe, HB, PES and amygdala (Figure 4a–c). Small immature or ramified DCX cells were noted within the superficial temporal cortex, as previously reported in Liu et al. (2008) and Srikandarajah et al. (2009) (Figure 4b) and in proximity to immature islands of cells of the paralaminar nucleus of the amygdala (Figures 3e and 4c) but without co‐expression for nestin. Mushashi^+^ multipolar cells and clusters were present in the SPL of TLobe (Figure 1l), SGZ and PVWM, a proportion of which were also nestin^+^ (Figure 4 d,e). Occasional small NEC co‐localized with NeuN (Figure 4f). **Astroglial markers**: Comparison of nestin to GFAP highlighted the restricted distribution of NEC compared with the dense astrogliosis (Figures 1a,b and 3b). Double labeling showed co‐expression of GFAP in a minor proportion of NEC (Figure 4g,h). GFAPδ labeling was more aligned with nestin, highlighting small multipolar cells in the SVZ, SGZ, hippocampal sulcus and, in particular, rows of cells in the PVWM near the ventricle (Figure 2n). Bipolar cells or elongated fibers were not observed with GFAP or GFAPδ however (Figure 4k). Double labeling confirmed GFAPδ expression in a proportion of NEC (Figure 4i,j). **Astroglial functional lineage markers**: Aq4 showed extensive labeling of glial processes in all regions, particularly around vessels (Figure 1j), in keeping with previous reports (Holen, 2011; Medici Frassoni, Tassi, Spreafico, & Garbelli, 2011). Double labeling highlighted populations of NEC in SPL, SGZ, and PVWM that were Aq4 immunonegative (Figure 4l,m). GS showed diffuse expression in astrocytic cells in the cortex and WM (Figure 1i); similar to Aq4 many NEC were GS immunonegative (Figure 4o,p) as were bipolar cells and long nestin^+^ fibers (Figure 4q). EAAT1 also showed labeling of astroglia in HS and TLobe (Figure 1k) but many NEC, including biopolar cells, appeared immunonegative (Figure 4r). Evidence of mTOR pathway activation using pS6 immunohistochemistry showed positive cytoplasmic expression in rows of small cells in the PHG WM, PVWM, SGZ, and in the HB sulcus, as well as small cells in layer I of the TLobe (Figure 1h). Double labeling of nestin with pS6 confirmed co‐localization in many of these small cells (Figure 4s). **Vascular markers**: Double labeling of nestin with vascular markers CD34 and PDGFRβ was carried out to explore the relationship between NEC and capillary vasculature. CD34 labeled only endothelium and expression in NEC, including bipolar cells, was not observed (Figure 4t,u). PDGFRβ highlighted pericytes and scattered small NG2‐like glial cells in the PVWM, SVZ, and SGZ as previously reported (Garbelli et al., 2015; Shepherd et al., 2013); there was occasional co‐expression of nestin in these cell types (Figure 4v).

### 3.5 Proliferative capacity of NEC

**Figure 5:**
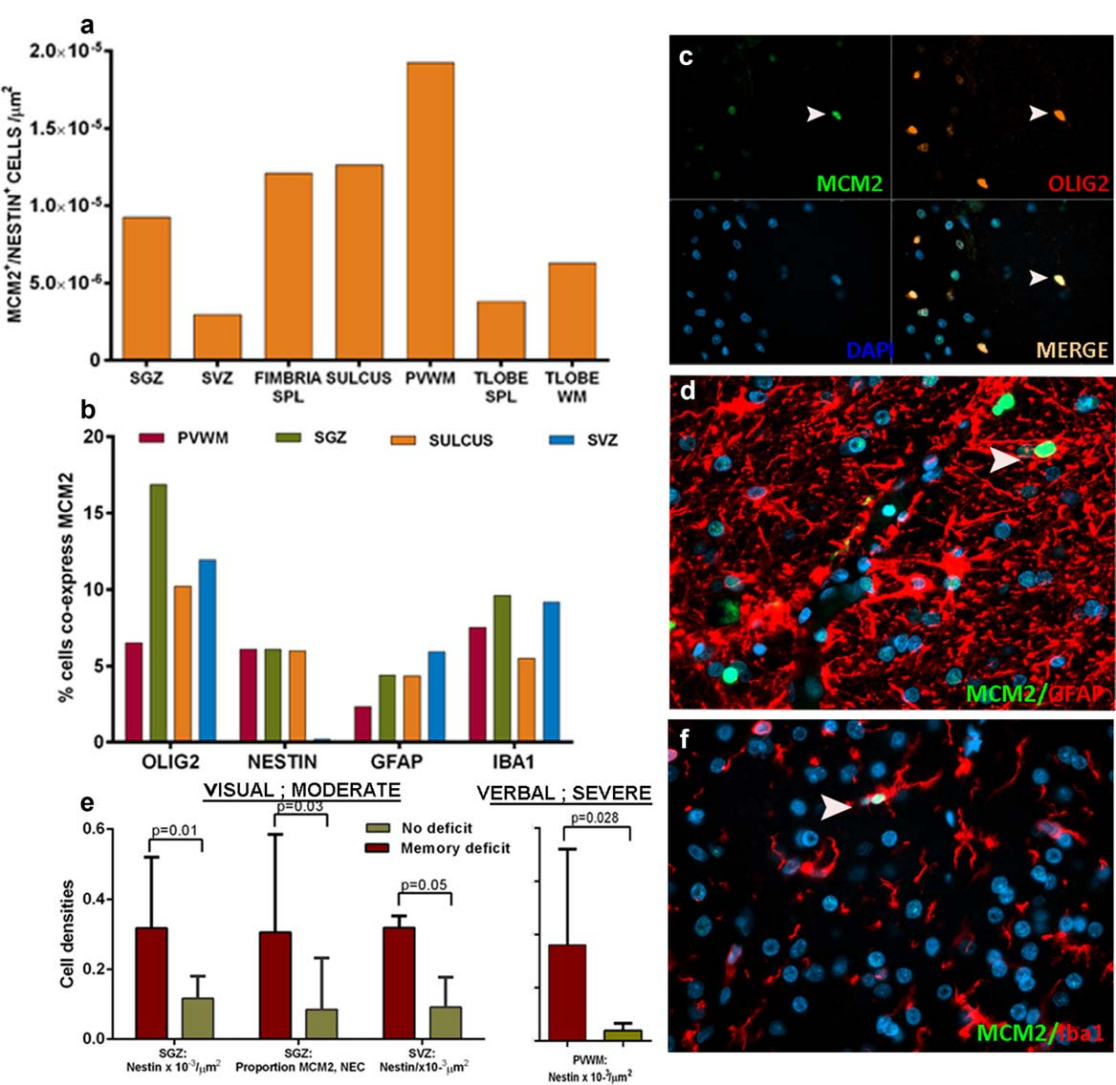
Proliferative fractions of NEC compared with other glial cell types. (a). Bar chart of the distribution of nestin/MCM2 double labeled cells in ROIs. This is shown for all regions in all epilepsy cases (with or without HS) showing variation between regions, with highest density in the PVWM (*p* = .014). These were significantly higher than non‐epilepsy control densities (not shown). (b) Bar chart showing relative contribution of proliferative fractions of NEC in hippocampal pool compared with other glial cell types. The percentage of nestin‐positive cells in all epilepsy cases in the HB, licensed for replication/MCM^+^ is compared with similar proliferative fractions of other glial lineages; oligodendroglial (Olig2+ cells), astroglia (GFAP^+^ cells), and microglia (Iba1^+^ cells) in three cases of HS (see Supporting Information Table S1). (c) Olig2/MCM2 with double labeled cell indicated with arrowhead. (d) GFAP/MCM2 with a double labeled cell indicated with arrowhead. (e) Bar chart of nestin cell densities in relation to the presence of a pre‐operative memory deficit. Significantly higher nestin cell densities were present in the SVZ and SGZ in patients with moderate visual memory deficits and in the PVWM in patients with severe verbal deficits (*p* < .05). The fractions of MCM^+^ cells co‐expressing nestin were higher in the SGZ in patients with moderate visual memory deficits (*p* < 0.05). (f) Iba1/MCM2 with a double‐labeled cell indicated with arrowhead

Quantitation showed greater densities of NEC in epilepsy compared with controls (*p* < .0001). In surgically epilepsy cases, significant variation in the density of NEC between TLobe zones was shown (*p* = .027), confirming semi‐quantitative observations. Double‐labeled Nestin^+^/MCM2^+^ cells showed highest density in the hippocampal PVWM, with significant variability between hippocampal regions (Figure 5a; *p* = .013). Nestin^+^/MCM2^+^ were more numerous in epilepsy cases than non‐epilepsy controls (*p* < .0001); Bipolar/radial glial‐like MCM2^+^ NEC were present in 56% of surgical epilepsy cases (Figure 2p,q) compared with 20% of controls. There was no significant difference in Nestin^+^/MCM2^+^ densities in any zones in cases with or without HS. Expressed as a proliferative fraction, 5.3% of NEC were MCM2^+^ in epilepsy cases. This was compared with MCM2^+^ proliferative fractions of other glial cell types, microglial (Iba1^+^; 7.9%) astroglia (GFAP^+^; 4.3%) and oligodendroglia (olig2^+^;11.4%) in similar regions (Figure 5 b–d,f). NEC, however, represented 29% of hippocampal MCM2^+^ cells compared with 11.4%/GFAP^+^, 25%/Iba1^+^, and 21%/olig2^+^.

### 3.6 NEC in cell culture

**Figure 6:**
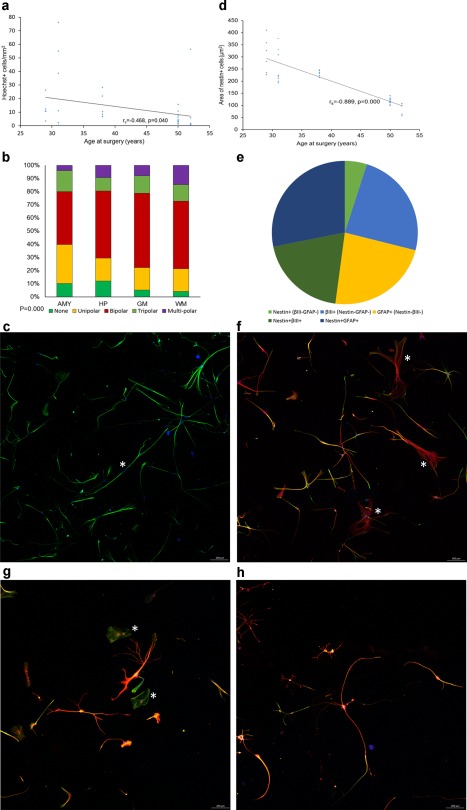
Immunophenotype and morphology of NEC in culture. (a) Scatterplot showing a slight decrease in the density of cells cultured as the age of patients at surgery increased (*p* = .040). Stacked bar chart (b) and an image (c) showing the varying proportions of uni‐, bi‐, tri‐, and multi‐polar NEC cultured from different brain regions (*p* = .000). NEC in C were derived from the hippocampus of 29‐years old patient with mesial TLE/HS (EAC3). *The longest aspect or length of this bipolar NEC was over 1500 µm. (d) Scatterplot showing the area of NEC cultured from all regions of all cases. Larger NEC were more likely to be cultured from resected brain samples of patients who were younger at the time of surgery (*p* = .000). (e) Pie chart showing that approximately half of the cells in culture were immune‐positive for nestin. The majority of NEC (green) were fate‐determined as neuronal or glial cells, and co‐expressed with βIII tubulin (f, red) or GFAP (g–h, red) respectively. (f) An image showing nestin and βIII tubulin immune‐positive cells with different degrees of primary branching from the hippocampus of patient EAC3. *These BIII tubulin cells have a larger soma than the neighboring bi/tri‐polar NEC, and expressed no or very little nestin protein. Images acquired from GFAP‐expressing NEC cultured from the hippocampus (g) and WM (h) of a 52‐years old patient with mesial TLE/HS (EAC6; GFAP, red; nestin, green). *NEC with no processes were more often observed in the amygdala and hippocampus, and they scarcely expressed GFAP. Both small and large multipolar cells expressing nestin and GFAP were commonly observed in the WM. Scale bar = 200 µm

Monolayer cell cultures were successfully derived from all resected specimens, and confluency of cultures (TPole) was generally reached around 3–4 weeks after cell dissociation (Figure 6a–h). The number of cells cultured was variable across regions and cases, with the most cells cultured from the gray matter (18 ±7 cells/mm^2^; Hoechst‐positive cells/mm^2^ ± *SEM*) and WM (13 ±5 cells/mm^2^) of the TLobe, and fewer cells from the hippocampus (9 ±3 cells/mm^2^), and the amygdala (2 ±0.47 cells/mm mm^2^). NEC were observed in cultures of all regions and cases. NEC were morphologically heterogeneous, composing of significantly more uni‐ and bi‐polar cells than tri‐ or multi‐polar cells in all regions of all cases (*p* = .000; Figure 6b,c,f). Generally, NEC with no processes were large cells, more frequently observed from the amygdala (10%) and hippocampus (12%; Figure 6g) compared with the gray (4%) or WM (5%). These NEC were usually only weakly positive for βIII tubulin or GFAP as shown in double label immunofluorescent studies (Figure 6g, asterisk). Multi‐polar cells with more than three primary branches were more commonly observed from the WM (15%; Figure 6h) than from other regions (amygdala, 4%; hippocampus, 9%, gray matter, 8%). The average size of NEC was similar between hippocampus (mean µm^2^ + *SEM*; 176 ± 19), gray matter (184 ± 27) and WM (190 ± 30), but NEC grown from the amygdala were noticeably larger with an average area of 282 ± 72 µm^2^.

The proportions of NEC that expressed βIII tubulin or GFAP were not significantly different between regions. On average across regions, 51% of cells cultured were NEC (Figure 6e), where 5 ± 1% expressed nestin only, 20 ± 3% co‐expressed βIII tubulin (Figure 6f) and 28 ± 3% co‐expressed GFAP (Figure 6g,h). The remaining cells were immunopositive for βIII tubulin (20 ± 2%) or GFAP (24 ± 4%) but immunonegative for nestin, and they often appeared larger than NEC.

### 3.7 Clinico‐pathological correlations

Semi‐quantitation of NEC in the PES hippocampus showed an inverse correlation with age of onset (*p* = .021) and a linear correlation with duration of epilepsy (*p* = .039); no significant differences were noted between pediatric and adult HS Type 1 cases. Quantitative analysis of hippocampal regions showed a positive correlation between age at surgery and mean Nestin^+^/μm^2^ (*p* = .03) and a trend for a negative correlation with the proliferative fraction (MCM2^+^) of NEC (*p* = .05). In culture, significantly more cells cultured from brain tissue of younger than older patients (*r*_s_ = −0.468, *p* = .040; Figure 6a). Furthermore, the average size of NEC negatively correlated with age (*r*_s_ = −0.889, *p* = .000; Figure 6d), with larger NEC grown from younger than older patients. There was no correlation between NEC and seizure types.

Moderate verbal and visual memory deficits were present in 6/15 and 6/15 and severe verbal and visual memory deficits in 2/15 and 4/15, respectively. Significantly higher densities of NEC in the amygdala were present in patients with moderate visual memory impairment and severe pre‐operative naming impairment (*p* < .05). Significantly higher nestin cell densities were present in the SVZ and SGZ in patients with moderate verbal and visual memory deficit and in the PVWM in patients with severe verbal deficit (*p* < .05) (Figure 5e). In addition increased Nestin^+^/MCM2^+^ cell densities were present in the SVZ in patients with moderate deficit and the fractions of MCM^+^ NEC were higher in the SGZ in patients with a moderate visual memory deficits (*p* < .05) (Figure 5e). There was no association between NEC and post‐surgical memory decline.

## 4 DISCUSSION

We have demonstrated a stereotypical pattern in the distribution and morphology of NEC in the human adult hippocampus and adjacent structures. NEC were consistently present, not only in the known neurogenic niches of the SGZ and SVZ, but also the SPL, fimbria, hippocampal sulcus and the PVWM of the PHG, identifying potential novel hippocampal niches for neurogenesis. The morphology of NEC varied with anatomical region and included bipolar cells and chains of NEC in the PVWM, which showed proliferative capacity and mTOR activation, but a relative lack of expression of mature astroglial functional markers such as Aq4 and GS. Morphologically similar NEC were grown *in vitro* and demonstrated glial and neuronal differentiation. As their distribution was reminiscent of that seen in the developing hippocampus, adult hippocampal NEC may represent sustained neuroglial migratory streams. Furthermore, NEC showed age‐related change and some association with memory dysfunction. It is conceivable that NEC populations, as a dynamic cell type, are relevant to hippocampal epileptogenesis and both acquired and developmental hippocampal abnormalities observed in epilepsy.

### 4.1 NEC in developmental and mature niches

Nestin is a marker of progenitor cells of both neuronal and glial lineage in the developing brain and has been widely used to distinguish undifferentiated from differentiated cell types (Kawaguchi et al., 2001; von Bohlen und Halbach, 2011). Previous studies have described bipolar cells with parallel processes extending from the SVZ inward into the developing hippocampus, declining in number between 14 and 22 weeks post‐conception (Yang et al., 2014). In five fetuses of up to 13 weeks gestation, we demonstrated NEC forming chains of cells and radial fibers, extending not only from the ventricular surface but also along the fimbria, the subpial surface of the hippocampus and developing hippocampal sulcus.

Studies in adult mammals and rodents traditionally recognize two main regions or niches for ongoing neurogenesis: the SGZ of the hippocampus, and the SVZ which gives rise to the rostral migratory stream (RMS) extending to the olfactory bulb. In rodents, the RMS comprises chains of neuroblasts, ensheathed by astrocytes (Brus, Keller, & Levy, 2013) and long nestin^+^ processes (Kamphuis, 2012). In the adult human brain, the existence of a RMS has been long debated and is considered likely to have diminished proliferative capacity (Brus et al., 2013; Curtis et al., 2007; Nogueira et al., 2014; Sanai et al., 2004; van Strien, van den Berge, & Hol, 2011). Vestigial structures around an extension of the lateral ventricle with migrating cells in chains, bipolar cells and thread‐like fibers have been reported (Curtis et al., 2007). In rodents, the hippocampal fissure, equivalent to the sulcus in humans, has also been identified as a niche and further potential source of progenitor cells in adult animals (Zhang et al., 2014). A further migratory stream has been described in mice extending from the hippocampal fimbra to dentate, composed of radial‐glial like cells, which persists into adulthood (Belmadani et al., 2015). Recent studies of human hippocampal development also identify the fimbria as a further source of progenitor cells for the dentate gyrus (Cipriani et al., 2015). These observations highlight the hippocampal formation as unique in both development and maturation with the close apposition of several neurogenic regions. Furthermore, these regions parallel the distribution of persistent NEC populations we observed in the adult human epileptic hippocampus.

### 4.2 Persistent radial glial in adult human brain

The persistence of NEC in the normal adult rat brain has been previously described (Hendrickson, Rao, Demerdash, & Kalil, 2011). Small multipolar NEC distributed throughout the forebrain, distinct from other astroglial, microglia, and oligodendroglial cell types (Hendrickson et al., 2011) compare with the small multipolar NEC we currently observe in the TLobe cortex, WM, and mesial structures. In rodent as well as human PM samples from the basal ganglia, small numbers of larger, NeuN co‐expressing NEC have been identified (Hendrickson et al., 2011); we observed similar cells but mainly localized in the amydgala. In adult PM tissues, NEC in the hippocampal SVZ, SPL, and fimbria, extending to hypothalamic structures, have previously been described, interpreted as persistent neurogenic streams (Nogueira et al., 2014). In TLE tissue, radial glial‐like cells, bipolar and astroglial like NEC have been reported in patients less than 14 years (Kruglyakova et al., 2005) and NEC in the SGZ SGZ in TLE patients <2 years (Blumcke et al., 2001).

In this study we demonstrated persistence of NEC in a stereotypical pattern. Striking observations included chains or rows of NEC in hippocampal, PHG WM, elongated nestin^+^ fibers and bipolar radial‐glial like NEC, often in proximity to the recess of the lateral ventricle; these features are suggestive of a residual/vestigial TLobe RMS. We also identified continuous streams of NEC extending from the fimbrial or supbial surface of the HB or pes to the dentate gyrus as well as surrounding the para‐laminar nucleus of the amygdala. Supportive data from our and previous *in vitro* studies (Arsenijevic et al., 2001; Roy et al., 2000) also demonstrate that NEC may be cultured from a number of brain regions including amygdala, hippocampus, temporal cortices, and the subcortical WM of mesial TLE/HS patients, even at the age of 52 years. Cultured cells showed a range of morphologies comparable to histology, including bipolar cells with long processes of >1 mm in culture, particularly from the TLobe and hippocampus. The elongated nestin^+^ fibers and bipolar cells are reminiscent of developmental radial glial fibers (Tabata, 2015). We consider these elongated NEC as unlikely to represent endothelial cells or pericytes as they occasionally showed terminal branches, varicosities and were negative for relevant vascular markers. Furthermore a regional predilection of these bipolar NEC to the temporal WM of the PHG and inferior TLobe WM was a consistent finding.

### 4.3 NEC as injury‐responsive progenitor cells

We also observed that bipolar NEC in the TLobe were licensed for replication. Proliferation of NEC types is well recognized at injury sites as part of the normal process of brain repair (Burda, Bernstein, & Sofroniew, 2016). An in vitro slice culture study in TLE patients following surgery showed an upregulation of NEC in relation to vessels (Verwer et al., 2015). In our previous study of the time course of NEC proliferation in relation to cortical injuries following depth electrode recordings, cells typically exhibited a radial‐glial like bipolar morphology and a close relationship to new‐formed vessels in acute lesions (Goc et al., 2014). Similar bipolar NEC have been reported in acute MS plaques (Moreels, Vandenabeele, Dumont, Robben, & Lambrichts, 2008). The acute influx of “injury‐responsive” NEC is argued as more likely to be from pre‐existing niches of NEC populations (Hendrickson et al., 2011) rather than recruitment from differentiated astrocytes, pericytes (ElAli, Theriault, & Rivest, 2014; Shin et al., 2013), or bone marrow‐derived stem cells (Borlongan, Glover, Tajiri, Kaneko, & Freeman, 2011). There is evidence that acute seizures can increase nestin expression (Jansson, Wennstrom, Johanson, & Tingstrom, 2009) and following status epilepticus, nestin^+^ radial glial fibers have been reported (Scorza et al., 2009). Quantitative studies comparing nestin expression in surgically resected TLE to PM control tissue in culture showed significantly higher levels in epilepsy cases (Verwer et al., 2015). We also demonstrated in our series significantly more NEC in epilepsy than controls. Although we cannot exclude other factors, including differences in fixation times and potentially anti‐epileptic drugs and other medications may influence staining and proliferation, our evidence supports that NEC are seizure‐responsive populations.

There is compelling evidence that seizures alter rates of hippocampal neurogenesis [reviewed in (Jessberger & Parent, 2015; Zhong et al., 2016)]. MCM2^+^ NEC were present in all zones in epilepsy cases and with significantly higher proliferative fractions of 6.6% compared with 3.3% in controls. This compares to our previous observations of proliferative fractions of 50% at acute injury sites to under 10% in chronic scars (Goc et al., 2014) suggesting a higher baseline activation of NEC in epilepsy as in previous studies (Verwer et al., 2015). The regions we noted with higher Nestin^+^/MCM2^+^ cell densities were the hippocampal sulcus and PVWM. Crespel et al. (2005), also reported frequent PCNA^+^ cells in the adult hippocampal sulcus in TLE, proposing this region as another neurogenic region. In another study MCM2^+^ was not noted in hippocampal NEC in ten HS cases but NG2+/Olig2+ cells were the most proliferative type (Geha et al., 2010). We were unable to achieve reliable NG2 immuolabeling, but confirmed that NEC contributed to the hippocampal proliferative pool, comparable to Iba1^+^, GFAP^+^, and Olig2^+^ glial cell types. Of note, bipolar NEC in the WM were MCM2^+^ in over half the epilepsy cases compared with only 20% of controls. Neural stem cells in the SVZ are derived from embryonic radial glial cells (Kriegstein & Alvarez‐Buylla, 2009); nestin^+^ radial glial with ongoing stem‐cell capacity have been previously reported in adult rat brain (Gubert, Zaverucha‐do‐Valle, Pimentel‐Coelho, Mendez‐Otero, & Santiago, 2009) and progenitor cells successfully isolated from the human adult WM (Lojewski et al., 2014). Our observations of a morphologically similar proliferating radial glial cell pool in the adult TLobe WM in epilepsy is therefore of potential relevance.

### 4.4 Differentiation of NEC and relevance to comorbidity and pathology in TLE

Studies in chronic TLE suggest a diminished capacity for neurogenesis (Marucci et al., 2013; Paradisi et al., 2010) which may result from a preferential switch of precursor cells to glial over neuronal differentiation (Hattiangady & Shetty, 2010). We explored the differentiation of NEC in both tissue sections and cell culture. Expression of neuronal markers (βIII tubulin and mushahsi) was noted but there was more evidence for glial maturation. Bipolar NEC exceptionally did not co‐express GFAP in sections, as previously noted (Kruglyakova et al., 2005), although GFAP expression was observed in cell culture. In contrast to a previous study (Nogueira et al., 2014) but in keeping with other reports (Dennie, Louboutin, & Strayer, 2016; Gao et al., 2016), we noted a virtual lack of co‐expression of nestin and DCX in immature populations in all regions suggesting distinct lineages or time points of cell expression. The distribution of NEC in both the TLobe and hippocampus showed similarity with GFAPδ (Martinian et al., 2009) which identifies subsets of specialized neurogenic astrocytes (Kamphuis et al., 2012; van den Berge et al., 2010). We also noted a paucity of labeling with GS, Aq4, and EAAT1, markers of mature, functioning astrocytes in many NEC. Furthermore, co‐expression of pS6 in hippocampal NEC supports mTOR pathway activation in these cells (Liu et al., 2014a *)*, also known to modulate glial cell function (Wang, Sha, Sun, Shen, & Xu, 2016). Hippocampal astrogliopathies are now considered central to pro‐epileptogenic mechanism in experimental models (Bedner et al., 2015; Kielbinski, Gzielo‐Jurek, & Soltys, 2016; Pekny et al., 2016). Our identification of NEC in the hippocampus, including CA1 in HS cases, as functionally immature, activated cells could be of pathophysiological relevance. Furthermore, glial proliferation following injury is also known to impair memory function (Sajja, Hlavac, & VandeVord, 2016); our identification of higher proliferative fractions of NEC in epilepsy patients with memory deficits, although only a small series, also warrants further investigation.

We noted the pattern of HS in the PES differed from the body, with preferential neuronal loss and gliosis in CA4 more than CA1 [Type 3 vs. Type 1 sclerosis in the ILAE classification system (Blumcke et al., 2013)]; this may relate to different distributions of injury responsive NEC. Furthermore, proliferative NEC with capacity and potential for glial/neuronal differentiation may contribute to the array of microscopic malformative and developmental tumors reported in TLobe in epilepsy (Supporting Information Figure S2).

In summary, NEC in the TLobe with ongoing proliferative capacity, likely represent the remnants of developmental migratory streams. NEC populations represent reactive cell populations in the adult with capacity for glial and neuronal differentiation and may be relevant to hippocampal epileptogenesis, histological alterations in TLE and co‐morbidities including memory decline.

## Supporting information

Supporting Information Figure 1.

Supporting Information Figure 2.

Supporting Information Table 1.

Supporting Information
