# Supplementary figures and images for: Nestin‐expressing cell types in the temporal lobe and hippocampus: Morphology, differentiation, and proliferative capacity
Source: Glia. 2017 Sep 19;66(1):62–77. doi: 10.1002/glia.23211 (PMC5724502; doi:10.1002/glia.23211)

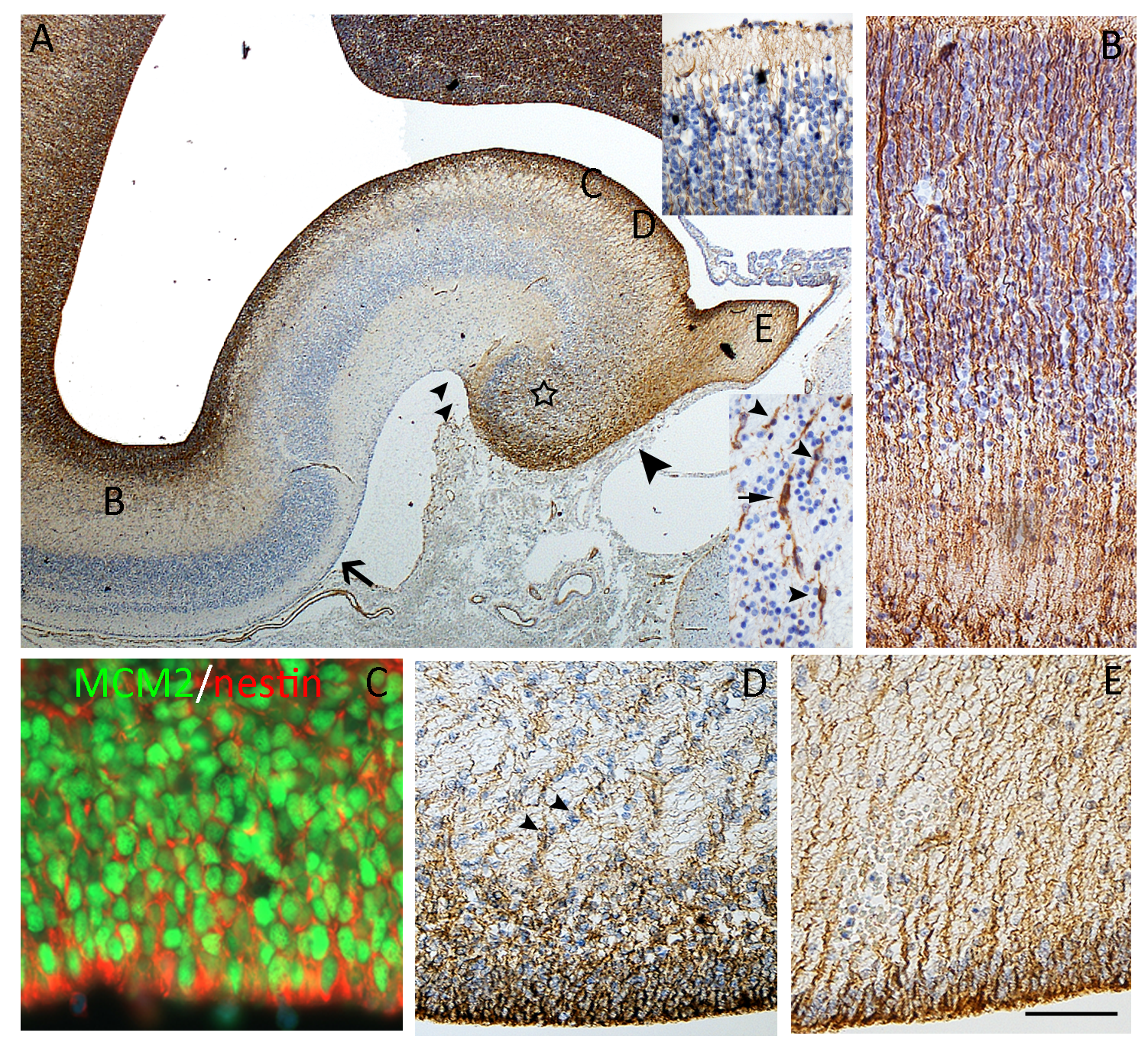

Supplement: Supplementary file 1 — Supporting Information Figure 1. [file GLIA-66-62-s001.tif]

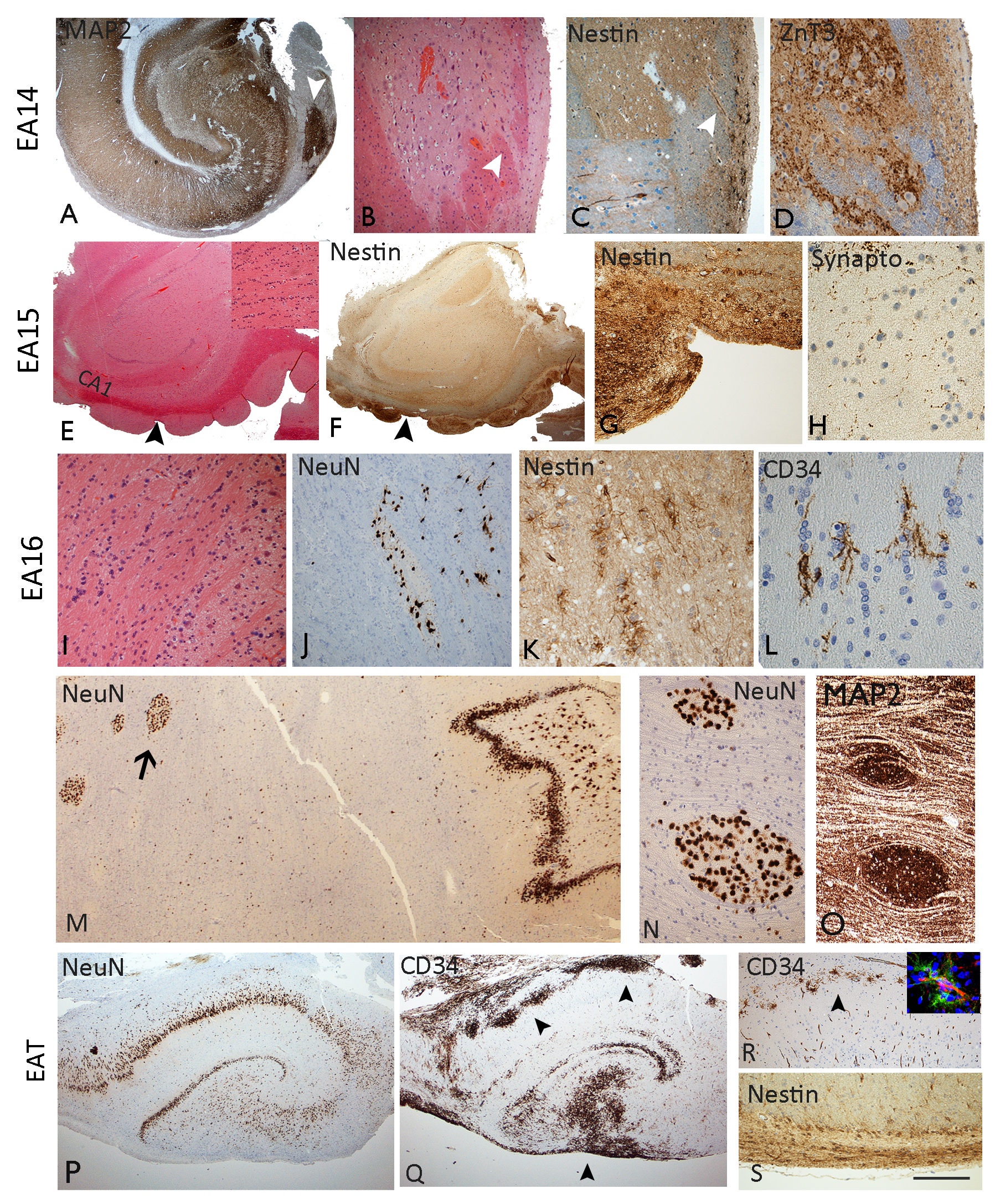

Supplement: Supplementary file 2 — Supporting Information Figure 2. [file GLIA-66-62-s002.tif]
