# Supplementary material for: Nestin‐expressing cell types in the temporal lobe and hippocampus: Morphology, differentiation, and proliferative capacity
Source: Glia. 2017 Sep 19;66(1):62–77. doi: 10.1002/glia.23211 (PMC5724502; doi:10.1002/glia.23211)
Supplement: Supplementary file 3 — Supporting Information Table 1. [file GLIA-66-62-s003.docx]

| **GROUP** | **CASE NUMBER** | **AGE OF ONSET OF EPILEPSY (Years)** | **AGE AT SURGERY (OR DEATH)**  **(Years)** | **GENDER** | **ILAE HS type IN BODY** | **Seizure type / syndrome** | **ILAE TYPE IN PES** | **Abnormal psychometry** | **REGIONS USED IN THIS STUDY** | **Analysis carried out** | **OTHER PATHOLOGY / CAUSE OF DEATH FOR PM CASES** |
| --- | --- | --- | --- | --- | --- | --- | --- | --- | --- | --- | --- |
| ADULT MTLE/HS TYPE 1 | EA1 | 6 | 42 | F | TYPE 1 | PS, CPS, GS | TYPE 1 | Pre-operative naming deficit i  Post-operative visual memory decline | HIPPOCAMPUS BODY, PES, AMYGDALA, PHG, TEMPORAL LOBE AND POLE | NESTIN-s/q  IF-q |  |
|  | EA2 | 17 | 36 | M | TYPE 1 | CPS, GS | TYPE 1 | No deficit | HIPPOCAMPUS BODY, PES, AMYGDALA, PHG, TEMPORAL LOBE AND POLE | NESTIN-s/q  IF -q |  |
|  | EA3 | 11 | 34 | M | TYPE 1 | PS, CPS, GS | TYPE 1 | No deficit | HIPPOCAMPUS BODY, PES, AMYGDALA, PHG, TEMPORAL LOBE AND POLE | NESTIN-s/q  IF –q  Glia/MCM2 |  |
|  | EA4 | 10 |  | F | TYPE 1 | PS, CPS | TYPE 3 |  | HIPPOCAMPUS BODY, PES, AMYGDALA, PHG, TEMPORAL LOBE AND POLE | NESTIN-s/q  IF-q  Glia/MCM2 |  |
|  | EA5 | 2 | 39 | M | TYPE 1 | PS, CPS, GS | TYPE 1 | Pre-operative verbal and visual memory deficit ; post-operative naming decline | HIPPOCAMPUS BODY, PES, AMYGDALA, PHG, TEMPORAL LOBE AND POLE | NESTIN-s/q  IF-q |  |
|  | EA6 | 3 | 54 | F | TYPE 1 | CPS, GS | INCOMPLETE | Pre-operative verbal and visual memory deficit | HIPPOCAMPUS BODY, PES, AMYGDALA, PHG, TEMPORAL LOBE AND POLE | NESTIN-s/q  IF-q |  |
|  | EA7 | 33 | 45 | F | TYPE 1 | CPS, GS | INCOMPLETE |  | HIPPOCAMPUS BODY, PES, AMYGDALA, PHG, TEMPORAL LOBE AND POLE | NESTIN-s/q  IF-q | MILD FCDIIIA (BLOCK 1C) |
|  | EA8 | 14 | 22 | F | TYPE 1 | CPS, GS | TYPE 1 | Pre-operative verbal and visual memory deficit | HIPPOCAMPUS BODY, PES, AMYGDALA, PHG, TEMPORAL LOBE AND POLE | NESTIN-s/q  IF-q | PREVIOUS INTRACRANIAL ELECTRODES |
|  | EA9 | 35 | 51 | M | TYPE 1 | CPS, GS | TYPE 3 | No deficit | HIPPOCAMPUS BODY, PES, AMYGDALA, PHG, TEMPORAL LOBE AND POLE | NESTIN-s/q  IF-q |  |
|  | EA10 | 31 | 45 | M | TYPE 1 | CPS. GS | TYPE 3 | Preoperative naming deficit  Post-operative decline in verbal memory and naming | HIPPOCAMPUS BODY, PES, AMYGDALA, PHG, TEMPORAL LOBE AND POLE | NESTIN-s/q  IF-q |  |
|  | EA11 | 3 | 53 | F | TYPE 1 | CPS, GS | INCOMPLETE | Pre-operative visual memory deficit | HIPPOCAMPUS BODY, PES, AMYGDALA, PHG, TEMPORAL LOBE AND POLE | NESTIN-s/q  IF-q  Glia/MCM2 |  |
|  | EA12 | 6 | 33 | M | TYPE 1 | CPS, GS | TYPE 3 | Pre-operative visual memory deficit | HIPPOCAMPUS BODY, PES, AMYGDALA, PHG, TEMPORAL LOBE AND POLE | NESTIN-s/q  IF-q |  |
|  | EA13 | 14 | 54 | F | TYPE 1 | CPS, GS | TYPE 1 |  | HIPPOCAMPUS BODY, PES, AMYGDALA, PHG, TEMPORAL LOBE AND POLE | NESTIN-s/q  IF | VARIABIITY IN PATTERN OF HS IN BODY |
|  | EA14 | 14 | 45 | M | No HS | GS | No HS |  | HIPPOCAMPUS BODY, PES, AMYGDALA, PHG, TEMPORAL LOBE AND POLE | NESTIN-QUALITATIVE | SMALL GANGLIOGLIOMA IN SUPERIOR TEMPORAL GYRUS |
|  | EA15 | 1.3 | 28 | F | TYPE 2 | AM |  |  | HIPPOCAMPUS BODY, PES, AMYGDALA, PHG, TEMPORAL LOBE AND POLE | NESTIN-QUALITATIVE |  |
|  | EA16 | 5 | 60 | M | TYPE 1 | AM |  |  | HIPPOCAMPUS BODY, PES, AMYGDALA, PHG, TEMPORAL LOBE AND POLE | NESTIN-QUALITATIVE  IF-q |  |

| ADULT MTLE/NO HS | EA17 | 20 | 35 | M | NO HS | CPS, GS | INCOMPLETE | Pre-operative verbal and visual memory deficit | HIPPOCAMPUS BODY, PES, AMYGDALA, PHG, TEMPORAL LOBE AND POLE | NESTIN-s/q  IF-q | PREVIOUS INTRACRANIAL ELECTRODES |
| --- | --- | --- | --- | --- | --- | --- | --- | --- | --- | --- | --- |
|  | EA18 | 19 | 24 | M | NO HS | PS, CPS, GS | NO HS | post-operative naming decline | HIPPOCAMPUS BODY, PES, AMYGDALA, PHG, TEMPORAL LOBE AND POLE | NESTIN-s/q  IF-q | MILD MCD IN TEMPORAL LOBE |
|  | EA19 | 9 | 28 | F | NO HS | PS, CPS | INCOMPLETE | post-operative naming decline | HIPPOCAMPUS BODY, PES, AMYGDALA, PHG, TEMPORAL LOBE AND POLE | NESTIN-s/q  IF-q | POSSIBLE LESION IN AMYGDALA ON MRI - NOT CONFIRMED IN PATHOLOGY |
|  | EA20 | 22 | 24 | M | NO HS | CPS, GS | TYPE 3 |  | HIPPOCAMPUS BODY, PES, AMYGDALA, PHG, TEMPORAL LOBE AND POLE | NESTIN-s/q  IF-q | MRI LESION IN AMYGDALA NOT CONFIRMED |
|  | EA21 | 15 | 30 | F | NO HS | CPS, GS | TYPE 1 | Pre-operative verbal memory deficit ; post-operative decline in verbal memory | HIPPOCAMPUS BODY, PES, AMYGDALA, PHG, TEMPORAL LOBE AND POLE | NESTIN-s/q | PREVIOUS INTRACRANIAL ELECTRODES |
|  | EA22 | 15 | 31 | M | NO HS | PS, CPS, GS | NO HS | Pre-operative naming deficit | HIPPOCAMPUS BODY, PES, AMYGDALA, PHG, TEMPORAL LOBE AND POLE | NESTIN-s/q  IF-q |  |
| PAEDIATRIC MTLE/HS TYPE 1 | EP1 | 4 | 8 | M | TYPE 1 | CPS | NOT EXAMINED | Not available | HIPPOCAMPUS BODY,  TEMPORAL LOBE | NESTIN-s/q  IF-q |  |
|  | EP2 | 0.6 | 14 | F | TYPE 1 | PS,SGS | NOT EXAMONED | Not available | HIPPOCAMPUS BODY,  TEMPORAL LOBE | NESTIN-s/q |  |
|  | EP3 | 5 | 11 | F | TYPE 1 | CPS | NOT EXAMINED | Not available | HIPPOCAMPUS BODY,  TEMPORAL LOBE | NESTIN-s/q |  |
|  | EP4 | 7.5 | 15 | M | TYPE 1 | PS,CPS,SGS | NOT EXAMINED | Not available | HIPPOCAMPUS BODY,  TEMPORAL LOBE | NESTIN-s/q |  |
|  | EP5 | 2.5 | 13 | M | TYPE 1 | CPS,SGS | NOT EXAMINED | Not available | HIPPOCAMPUS BODY,  TEMPORAL LOBE | NESTIN-s/q |  |
| ADULT MTLE CELL CULTURE ONLY | EAC1 | 8 | 50 | M | TYPE 1 | AM, GS | TYPE 1 | Not available | HIPPOCAMPUS, TEMPORAL LOBE | CELL CULTURE STUDY ONLY |  |
|  | EAC2 | 6 | 38 | F | TYPE 1 | AM | INCOMPLETE | Not available | HIPPOCAMPUS, TEMPORAL LOBE | CELL CULTURE STUDY ONLY |  |
|  | EAC3 | 15 | 29 | M | TYPE 2 | CPS,SGS | TYPE 1 | Not available | HIPPOCAMPUS, TEMPORAL LOBE | CELL CULTURE STUDY ONLY |  |
|  | EAC4 | 4 | 31 | F | TYPE 1 | CPS | TYPE 3 | Not available | HIPPOCAMPUS, TEMPORAL LOBE, AMYGDALA | CELL CULTURE STUDY ONLY |  |
|  | EAC5 | 2 | 50 | M | TYPE 1 | AM | INCOMPLETE | Not available | HIPPOCAMPUS, TEMPORAL LOBE, AMYGDALA | CELL CULTURE STUDY ONLY | PREVIOUS INTRACRANIAL ELECTRODES (10 months) |
|  | EAC6 | 2 | 52 | F | TYPE 1 | CPS | TYPE 3 | Not available | HIPPOCAMPUS, TEMPORAL LOBE | CELL CULTURE STUDY ONLY | PREVIOUS INTRACRANIAL ELECTRODES (12 months) |
| ADULT MTLE LEAT | EAT1 | 12 | 27 | F | TYPE 1 |  | NO HS |  | HIPPOCAMPUS BODY, PES, TEMPORAL LOBE | QUALITATIVE |  |
|  | EAT2  14-1937 | 20 | 25 | M | TYPE 2 | AM | NO HS |  | HIPPOCAMPUS BODY, PES, TEMPORAL LOBE | QUALITATIVE |  |
|  | EAT3  16-188 | 7 | 46 | F | TYPE 3 | AM | TYPE 3 |  | HIPPOCAMPUS BODY, PES, TEMPORAL LOBE | QUALITATIVE |  |
| POST MORTEM - EPILEPSY | EPM1 | 4 | 72 | M | TYPE 1  BILATERAL | FS, CPS, GS  SYNDROME : SYMPTOMATIC/FOCAL EPILEPSY | NOT EXAMINED | Not available | HIPPOCAMPUS BODY (BOTH SIDES) | NESTIN-s/q | CVD |
|  | EPM2 | 4 | 26 | M | TYPE 1  BILATERAL | AbS, GTCS  SYNDROME : SYMPTOMATIC/FOCAL EPILEPSY | NOT EXAMINED | Not available | HIPPOCAMPUS BODY (BOTH SIDES) | NESTIN-s/q | CONGENITAL HEART DISEASE |
|  | EPM3 | childhood | 47 | F | TYPE 1  BILATERAL | GTCS  SYNDROME : U/C | NOT EXAMINED | Not available | HIPPOCAMPUS BODY (BOTH SIDES) | NESTIN-s/q | CARDIAC DISEASE |
|  | EPM4 | 10 MONTHS | 71 | F | TYPE 1  BILATERAL | GTCS  SYNDROME : U/C | NOT EXAMINED | Not available | HIPPOCAMPUS BODY (BOTH SIDES) | NESTIN-s/q | PE |
|  | EPM5 | Neonatal | 76 | F | TYPE 1 | PS, GS, MJ  SYNDROME : U/C | NOT EXAMINED | Not available | HIPPOCAMPUS  BODY | NESTIN-s/q | BRONCHOPNEUMONIA |
|  | EPM6 | 21 | 55 | M | TYPE 1 | CPS. , SPS,  SYNDROME : TLE | NOT EXAMINED | Not available | HIPPOCAMPUS  BODY | NESTIN-s/q | LIMITED POST MORTEM TO HEAD ONLY EXAMINATION |
| CONTROL PM | C1 | NA | 41 | F | NO HS | - | NOT EXAMINED | Not available | HIPPOCAMPUS BODY (BOTH SIDES) | NESTIN-s/q | UK |
|  | C2 | NA | 58 | F | NO HS | - | NOT EXAMINED | Not available | HIPPOCAMPAL BODY | NESTIN-s/q | IHD |
|  | C3 | NA | 57 | F | NO HS | - | NOT EXAMINED | Not available | HIPPOCAMPAL BODY | NESTIN-s/q | PANCREATITIS |
|  | C4 | NA | 75 | M | NO HS | - | NOT EXAMINED | Not available | HIPPOCAMPUS BODY (BOTH SIDES) | NESTIN-s/q | UK |
|  | C5 | NA | 28 | M | NO HS | - | NOT EXAMIINED | Not available | HIPPOCAMPUS BODY (BOTH SIDES),TEMPORAL CORTEX, AMYGDALA | NESTIN-s/q | CARDIAC DEATH |
|  | C6 | NA | 33 | M | NO HS | - | NOT EXAMINED | Not available | HIPPOCAMPUS BODY | NESTIN-s/q | UK |
|  | C7 | NA | 36 | M | NO HS | - | NOT EXAMINED | Not available | HIPPOCAMPUS BODY | NESTIN-s/q | UK |
|  | C8 | NA | 38 | F | NO HS | - | NOT EXAMINED | Not available | HIPPOCAMPUS BODY (BOTH SIDES) | NESTIN-s/q | UK |
|  | C9 | NA | 61 | F | NO HS | - | NOT EXAMINED | Not available | HIPPOCAMPUS BODY (BOTH SIDES) | NESTIN-s/q | UK |
|  | C10 | NA | 56 | M | NO HS | - | NOT EXAMINED | Not available | HIPPOCAMPUS BODY, AMYGDALA , TEMPORAL LOBE | NESTIN-s/q | IHD |
|  | C11 | NA | 57 | M | NO HS | - | NOT EXAMINED | Not available | HIPPOCAMPUS BODY, AMYGDALA , TEMPORAL LOBE | NESTIN-s/q | IHD |
|  | C12 | NA | 49 | M | NO HS | - | NOT EXAMINED | Not available | HIPPOCAMPUS BODY, AMYGDALA , TEMPORAL LOBE | NESTIN-s/q | IHD |
|  | C13 | NA | 48 | M | NO HS | - | NOT EXAMINED | Not available | HIPPOCAMPUS BODY, AMYGDALA , TEMPORAL LOBE | NESTIN-s/q | IHD |
|  | C14 | NA | 44 | M | NO HS | - | NOT EXAMINED | Not available | HIPPOCAMPUS BODY, AMYGDALA , TEMPORAL LOBE | NESTIN-s/q | BRONCHOPNEUMONIA ; IHD |
|  | c15 | NA | 62 | F | NO HS | - | NOT EXAMINED | Not available | HIPPOCAMPUS BODY, AMYGDALA | Nestin-s/q | ADENOID CYSTIC CARCINOMA |

**Supplemental Table 1. Detail of each case used in the study including clinical and psychometric data and the type of study carried out.** The cases are numbered with the following nomenclature : EA = epilepsy adult surgical, EP=epilepsy paediatric surgical, EPM=epilepsy post-mortem tissue, C = control post-mortem tissue. Seizure types have been recorded using the nomenclature on their records. PS= partial seizures, CPS= complex partial seizures, GS= generalised seizures including secondary generalised seizures, GTCS = generalised tonic-clonic seizures, AM = automotor seizures, AbS = absence seizures, MJ=myoclonic jerks, FS = febrile seizures. TLE = temporal lobe epilepsy, RE= refractory epilepsy no further detail of seizures, U/C = epilepsy syndrome unclassified, HS= hippocampal sclerosis, IF=Immunofluoresence, PM = post mortem, s/q=semi-quantitative, q=quantitative, Glia/MCM2 = the study of olig2, GFAP or Iba1 proliferative fractions, LEAT = long-term epilepsy-associated tumour, IHD = ischaemic heart disease, UK= unknown, CVD = cerebrovascular disease, PE = pulmonary embolus. The developmental controls are not included in this list.
