# Supplementary material for: Nestin‐expressing cell types in the temporal lobe and hippocampus: Morphology, differentiation, and proliferative capacity
Source: Glia. 2017 Sep 19;66(1):62–77. doi: 10.1002/glia.23211 (PMC5724502; doi:10.1002/glia.23211)
Supplement: Supplementary file 4 — Supporting Information [file GLIA-66-62-s004.docx]

Supplementary Methods

**QUANTITATIVE ANALYSIS OF DOUBLE LABELLED CELLS**

In 16 surgical cases (15 adult and 1 pediatric surgical cases with and without HS) and 5 PM controls (see Supplementary Table 1), MCM2/nestin double-labelled sections from HB, PES and Tlobe were quantified. Serial sequential images were captured using 40x objective from the following ROI: TLobe SPL, Tlobe WM, SGZ, SVZ, FZ/SPL, hippocampal sulcus, and PVWM (mean area per zone 3.8 x 10^5^ μm^2^ (range 0.3 to 8.6 x 10^5^ μm^2^) using Zen Blue software and Zeiss IF microscope (Zeiss, Göttingen, Germany). The densities of single and double-labelled cells were quantified, with care to exclude nestin- immunopositive endothelial cells, and expressed per unit area. In three type 1 HS cases (EA3, EA4 and EA7) single and double-labelled cells for MCM2 with olig2, GFAP or Iba1 co-expression in HB ROI were similarly quantified to characterize the relative proportions of MCM2 immunopositive proliferating cells.

**IMMUNOFLUORESCENT LABELLING**

Sections were incubated in a solution containing anti-nestin antibodies diluted in DAKO REAL antibody diluent (1:1000; DAKO, UK) overnight at 4 ºC, and on the next day, anti-mouse HRP secondary solution (Vector Laboratories Inc., Peterborough, UK) was applied for 30 minutes, before fluorescein-labelled antibody in tyramide signal amplification (TSA) buffer (1:500, Perkin Elmer, Massachusetts, USA) was applied for eight minutes. Sections were thoroughly washed using phosphate buffer saline (PBS), and then immersed in 0.9% hydrogen peroxide solution and 10 % normal horse serum in PBS for ten minutes. The secondary primary antibodies were applied (Table 2) overnight at 4 ºC. On the following day, species-specific secondary HRP antibodies was applied for 30 mins, before rhodamine-labelled antibody in TSA buffer was applied for eight minutes. Sections were coverslipped using DAPI mounting medium (Vector Laboratories Inc., Peterborough, UK). Qualitative examinations of double-labeled sections was carried out with (Zeiss Axio Imager Z2), and confocal laser scanning microscopes (LSM-Meta 710, Zeiss, Göttingen, Germany). The software, Zen 2012 blue lite (Zeiss, Göttingen, Germany), was used to view z-stacks and tile of confocal images.

**CELL CULTURE**

For in vitro studies, 0.5 to 1.2 grams of fresh tissue were sampled from the hippocampus (pes and hippocampal body), and grey and white matter of the temporal pole of six surgical patients with TLE/HS (cases EAC1-6). 0.2 grams of tissue from amygdala from two cases (EAC4-5) were also cultured. Fresh tissue were collected immediately after surgery and were washed in Hanks balanced salt solution (HBSS; Ca2+ and Mg2+ free; Gibco, Thermo Fisher Scientific, Hemel Hempstead, UK) to remove excess blood. Visible blood vessels, meninges, and any necrotic tissue were macroscopically dissected. Tissue was homogenised using GentleMacs automated dissociator and reagents available in the MACS neural dissociation kit (catalogue no. 130-093-231) following manufacturer’s protocol (Miltenyl Biotechnology, Surrey, UK). Cells were filtered through a 100µm strainer (Fisher Scientific UK, Loughborough, UK), and were centrifuged for ten minutes before the pellet was resuspended in MACS Neuro Medium (Miltenyl Biotechnology, Surrey, UK) supplemented with 1% MACS NeuroBrew-21, 1% penicillin and streptomycin, heparin sulfate, 10 ng/mL EGF, 10 ng/mL bFGF, and 10% fetal bovine serum (Gibco, Thermo Fisher Scientific). Cell suspension was quantified using the Sceptor 2.0 automated cell counter (EMD Millipore, Hertfordshire, UK), and cells were plated between 1x10^4^ to 10^5^ cells/mL per well in sterile, cell culture-treated six-wells plates (Nunclon; Thermo Fisher Scientific). Cells were incubated in a 95%O2/5% CO2 incubator at 37ºC for 48 hours. Debris were removed, and cells were replaced with fresh medium and cultured in the incubator for four weeks. Medium was replaced every three days. For immunocytochemistry, cells were fixed in 4% paraformaldehyde solutions (Santa Cruz Biotechnology, Gemany) for 20 mins, and incubated in 0.3% triton solutions for ten mins, and in blocking solution consisting of 10% normal goat serum (NGS; Sigma Aldrich, ) for ten mins. Solutions consisting of monoclonal anti-nestin (1:500) and polyclonal GFAP (1:500) or βIII tubulin antibodies (1:600) diluted in 10% NGS were applied to the cells overnight at 4ºC. On the following day, Alexa Fluor 488-conjugated anti-mouse and Alexa Fluor 546-conjugated anti-rabbit secondary antibodies (1:400) diluted in 10% NGS were added to the preparation for 1.5 hour at room temperature, and then a solution consisting of hoechst in PBS was applied for 15 mins. Immunolabelled cells were visualized using confocal laser microscopy and an averaged sampled area of 45mm^2^ (or 13 cells/mm^2^) were imaged per region per case for quantification. Images were imported into the image analysis software, Definiens Tissue Studio 3.6 and Definiens Developer x64 (Definiens AG; Munich, Germany), for automated quantification as previously described (Liu et al., 2014). In brief, images were imported into Tissue Studio 3.6, and after appropriate tissue background separation, hoechst-positive nuclei detection, and nestin and GFAP/ βIII tubulin immunopositive labelling were optimally thresholded according to staining intensity at 1.32, 28 and 45 respectively. Processed images were then analysed using Definiens Developer. For each image, the software calculated the number and size (µm^2^) of hoechst-positive nuclei, the area (µm^2^) of nestin, GFAP/ βIII tubulin or colocalised labelling around each hoechst-positive nucleus, and the number of hoechst-positive cells with nestin, GFAP/ βIII tubulin or colocalised immunolabelling. For each region of each case, final results were expressed as percentages of NEC that coexpressed GFAP or βIII-tubulin, percentages of hoechst-positive cells that expressed nestin, GFAP or βIII-tubulin, or both. The average area of NEC or GFAP/ βIII-tubulin cells from each region/case were also noted.

**PSYCHOMETRY METHODS FOR EPILEPSY SURGICAL PATIENTS**

In brief, pre-operative cognitive data was compared to retest data at one year following surgery, as previously described (Thompson *et al.*, 2015) and were available for the majority of patients from routine surgical evaluations undertaken at the National Hospital. Memory was assessed with the List Learning and Design Learning subtests from the Adult Memory & Information Processing Battery (AMIPB) prior to 2007 and its successor the BIRT Memory and Information Processing Battery (BIMPB) from 2007. These measures have previously been shown to be sensitive to hippocampal pathology (2). Naming capacity was assessed using the Graded Naming Test (GNT) (Bird & Cipolotti, 2007), a measure sensitive to dominant temporal lobe resections (Bonelli *et al.*, 2012). Pre-operatively patients were classified as having a moderate verbal or visual memory deficit if they scored 1 SD or more below average, and as having a severe deficit if they scored 2 SD or more below average. A patient was classified as having experienced a post-operative decline in verbal memory, visual memory and naming capacity if their post-operative scores declined more than would be expected from retesting on the basis of the reliable change indices (with a confidence interval of 90%) (Bird *et al.*, 2004, Thompson 2015).

Baxendale, S., Thompson, P., Harkness, W. & Duncan, J. (2006) Predicting memory decline following epilepsy surgery: a multivariate approach. *Epilepsia*, **47**, 1887-1894.

Bird, C.M. & Cipolotti, L. (2007) The utility of the recognition memory test and the graded naming test for monitoring neurological patients. *The British journal of clinical psychology / the British Psychological Society*, **46**, 223-234.

Bird, C.M., Papadopoulou, K., Ricciardelli, P., Rossor, M.N. & Cipolotti, L. (2004) Monitoring cognitive changes: psychometric properties of six cognitive tests. *The British journal of clinical psychology / the British Psychological Society*, **43**, 197-210.

Bonelli, S.B., Thompson, P.J., Yogarajah, M., Vollmar, C., Powell, R.H., Symms, M.R., McEvoy, A.W., Micallef, C., Koepp, M.J. & Duncan, J.S. (2012) Imaging language networks before and after anterior temporal lobe resection: results of a longitudinal fMRI study. *Epilepsia*, **53**, 639-650.

Thompson, P.J., Baxendale, S.A., McEvoy, A.W. & Duncan, J.S. (2015) Cognitive outcomes of temporal lobe epilepsy surgery in older patients. *Seizure : the journal of the British Epilepsy Association*, **29**, 41-45.
